# Antioxidant Nanohybrid Materials Derived via Olive Leaf Extract Incorporation in Layered Double Hydroxide: Preparation, Characterization, and Evaluation for Applications

**DOI:** 10.3390/antiox14081010

**Published:** 2025-08-18

**Authors:** Achilleas Kechagias, Areti A. Leontiou, Alexios Vardakas, Panagiotis Stathopoulos, Maria Xenaki, Panagiota Stathopoulou, Charalampos Proestos, Emmanuel P. Giannelis, Nikolaos Chalmpes, Constantinos E. Salmas, Aris E. Giannakas

**Affiliations:** 1 Department of Food Science and Technology, University of Patras, 30100 Agrinio, Greece; up1110842@upatras.gr (A.K.); aleontiu@upatras.gr (A.A.L.); alexvard@upatras.gr (A.V.); 2 GAEA Products S.M. S.A., 1st km Agriniou-Karpenissiou National Rd., 30100 Agrinio, Greece; 3 Division of Pharmacognosy and Natural Products Chemistry, Department of Pharmacy, National and Kapodistrian University of Athens, 15771 Athens, Greece; stathopan@pharm.uoa.gr; 4 PharmaGnose S.A., Papathanasiou 24, 34100 Chalkida, Greece; maxenaki@pharm.uoa.gr; 5 Department of Sustainable Agriculture, University of Patras, 30100 Agrinio, Greece; panstath@upatras.gr; 6 Laboratory of Food Chemistry, Department of Chemistry, National and Kapodistrian University of Athens, Zografou, 15771 Athens, Greece; harpro@chem.uoa.gr; 7 Department of Materials Science and Engineering, Cornell University, Ithaca, NY 14850, USA; epg2@cornell.edu; 8 Department of Material Science and Engineering, University of Ioannina, 45110 Ioannina, Greece

**Keywords:** olive leaf extract, layered double hydroxide, antioxidant nanohybrids, total polyphenol content, enzymatic assisted extraction, 3 hydroxytyrosol, oleuropein, luteolin-7-o-glucoside, apigenin-4-o-glucoside

## Abstract

In this study, an innovative and sustainable strategy for the valorization of olive leaves, an underutilized agro-industrial byproduct, was developed through enzymatic-assisted aqueous extraction to produce a polyphenol-rich olive leaf extract (OLE). The extract contained notable concentrations of hydroxytyrosol (0.53 mg/L), luteolin-7-o-glucoside (0.70 mg/L), apigenin-4-o-glucoside (0.18 mg/L), and oleuropein (4.24 mg/L). For the first time, this OLE was successfully nanoencapsulated into layered double hydroxides (LDHs) synthesized at Zn^2+^/Al^3+^ molar ratios of 1:1, 2:1, and 3:1, resulting in a series of OLE@LDH_Zn/Al_x/1 nanohybrids. Comprehensive structural characterization confirmed the successful intercalation of OLE within the LDH interlayer galleries. Antioxidant activity (via DPPH assay), total polyphenol content (TPC), and antibacterial tests were conducted to evaluate functionality. Among the nanohybrids, OLE@LDH_Zn/Al_1/1 exhibited the highest TPC (606.6 ± 7.0 mg GAE/L), the lowest EC_50,DPPH_, EC_50,ABTS_, and EC_50,FRAP_ values (27.88 ± 1.82, 25.70 ± 0.76, and 39.42 ± 2.16 mg/mL), and superior antibacterial performance against *E. coli* and *S. aureus*. Moreover, pH-dependent release revealed targeted polyphenol release under acidic conditions (pH = 1), simulating gastric environments. These results highlight LDHs, particularly with a Zn/Al ratio of 1:1, as promising nanocarriers for the stabilization and controlled release of plant-derived polar phenols, with potential applications in nutrition, food preservation, and biomedicine.

## 1. Introduction

In recent years, global trends in bioeconomy and sustainability have motivated scientists and researchers to develop “green” technologies aimed at reducing carbon dioxide emissions, minimizing dependence on fossil fuels, and decreasing food waste [1,2,3,4,5]. In alignment with these priorities, biomass valorization, the utilization of food and agricultural byproducts and side streams, and the reduction of food waste have become key areas of scientific focus [6,7,8,9,10,11,12]. In the field of Food Technology, these efforts are primarily directed toward the recovery of biopolymers from biomass as sustainable alternatives to fossil fuel-derived plastics, as well as the extraction of bioactive compounds for use in functional foods and bio-based preservatives [9,13,14]. Among the most prominent categories of bioactive compounds are natural extracts and essential oils (EOs) [15,16,17,18,19,20] which exhibit antioxidant, antibacterial, antifungal, and anticancer properties, rendering them highly promising for applications in food preservation and nutrition [15,18,19,20]. To enhance their efficacy and control their release, encapsulation within cost-effective nanocarriers such as layered silicates and natural zeolites has been proposed [20,21,22,23,24,25].

Layered silicates include naturally abundant nanoclays such as montmorillonite and halloysite, as well as synthetically produced LDHs. The key distinction between these materials lies in their ion exchange capabilities: LDHs preferentially intercalate negatively charged ions, whereas natural nanoclays typically intercalate positively charged cations [26,27,28]. Both LDHs and nanoclays have been widely studied in recent years as nanocarriers for bioactive compounds [25,29,30]. LDHs, which are two-dimensional (2D) clay materials of the hydrotalcite type, are particularly well suited for intercalating bioactive molecules within their interlayer spaces [25]. They can be readily dispersed into polymeric matrices, enabling the incorporation of specific bioactive compounds such as flavonoids (e.g., quercetin), phenolic acids (e.g., gallic acid and caffeic acid), and other plant-derived antioxidants.

LDHs offer numerous advantages, including low cost, high biocompatibility, non-toxicity, pH-dependent stability, controlled release behavior, high anion exchange capacity, low surface charge, and excellent thermal and chemical stability [25,31]. Various bioactive anions such as salicylate, d-gluconate, citrate, l-ascorbate, and cinnamate have been successfully intercalated into LDHs to form LDH/bioactive hybrids with antioxidant and antibacterial properties, suitable for use in food preservation either directly or embedded in polymeric or biopolymeric matrices [32,33,34,35,36,37,38,39,40]. To the best of our knowledge, polar phenols derived from olive oil or olive tree leaves have not yet been reported as intercalated compounds in LDHs. Encapsulation of such biologically active molecules within LDH layers may act as a protective “chemical flask jacket”, shielding them from biodegradation while enhancing their cellular uptake [41].

Olive tree leaves, a waste of olive oil production, are typically discarded despite being a rich source of bioactive compounds [42,43]. These leaves contain high concentrations of valuable polar phenols including hydroxytyrosol (HT), oleuropein (oleur), luteolin-7-o-glucoside (lut-7-ο-glu), and apigenin-4-o-glucoside (apig-4-o-glu). Recovering these health-promoting compounds supports their application in cosmetics, biomedicine, and the food industry as dietary supplements or natural additives [42]. Recent studies have demonstrated the successful valorization of olive tree leaves using environmentally friendly extraction techniques, such as enzyme-assisted methods, yielding OLE with a total polyphenol content of up to 605.55 mg GAE/L and high antioxidant activity [44].

Herein, we report for the first time the successful intercalation and encapsulation of oleuropein-rich olive leaf extract (OLE) into Zn–Al-based layered double hydroxides (LDH_Zn/Al). LDHs were synthesized using three different Zn^2+^:Al^3+^ molar ratios (1:1, 2:1, and 3:1), producing materials referred to as LDH_Zn/Al_1_1, LDH_Zn/Al_2_1, and LDH_Zn/Al_3_1, respectively. The resulting nanohybrids, OLE@LDH_Zn/Al_x_1 (x = 3, 2, 1), were thoroughly characterized by X-ray diffraction (XRD), Fourier transform infrared (FTIR) spectroscopy, high-resolution scanning electron microscopy (HR-SEM), and nitrogen porosimetry. The antioxidant activity of the OLE@LDH_Zn/Al nanohybrids was evaluated using the 2,2-diphenyl-1-picrylhydrazyl (DPPH) assay. Antibacterial activity was tested against *Escherichia coli* and *Staphylococcus aureus*, and controlled-release experiments were performed to assess the release profile of the hybrids. This comprehensive study offers new insights into selecting optimum hybrid compositions for potential applications in nutrition and biomedicine as controlled-release nanocarriers for bioactive polar phenols.

## 2. Materials and Methods

### 2.1. Materials

Zn(NO_3_)_2_·6H_2_O, Al(NO_3_)_3_·9H_2_O, and NaOH were purchased from Sigma Aldrich (Darmstadt, Germany). For analytical purposes, 2,2-diphenyl-1-picrylhydrazyl (DPPH·), 2,2-azino-bis(3-ethylbenzothiazoline-6-sulfonic acid) diammonium salt, hydrogen peroxide, 2,4,6-tripyridy-s-triazine (TPTZ), FeCl_3_, hydrochloric acid (37%), buffer (CH_3_COONa·3H_2_O), and Folin–Ciocalteu reagent were purchased from Sigma Aldrich (Darmstadt, Germany), and gallic acid (also known as 3,4,5-trihydroxybenzoic acid) 99% was isolated from Rhus chinensis Mill (JNK Tech. Co., Seongnam, Republic of Korea).

All solvents were HPLC grade; acetonitrile (99.9% purity), water (≥99.9% purity), and methanol (>99.8% purity) were obtained from ChemLab (Zeldegem, Belgium). The HT reference standard was purchased from ExtraSynthase (Lyon Nord, France), while the lut-7-o-glu, apig-4-o-glu, and oleuropein reference standards were purchased from Sigma Aldrich (Darmstadt, Germany). Olive (olea europaea) leaves, of the koroneiki variety harvested in the year 2023, were obtained from a producer in the region of Agrinio, Greece. The leaves were dried at 50 °C for 5 h.

### 2.2. Enzymatic-Assisted Extraction

The enzymatic-assisted extraction of OLE was conducted according to our recent study [40]. In detail, the following commercial enzyme preparations were used—the pectinolytic preparation Pectinex XXL and Viscozyme L (containing cellulase, hemicellulase, and xylanase), both obtained from Novozymes A/S (Bagsvaerd, Denmark). Finely ground olive leaves (particle size < 700 μm) were mixed with water at a ratio of 10:1 (*v*/*w*), acidified to pH 4.0 with HCl, and left to rehydrate for 1 h at 25 °C. Following pH adjustment (pH 4.0), a 100.0 g portion of the suspension was placed in a water bath at 50 °C (Memmert Schutzart DIN 40,050-IP 20, Büchenbach, Germany) for 20 min prior to enzyme addition (Pectinex XXL and Viscozyme L). After incubation at 50 °C, the sample was transferred to a boiling water bath for 10 min to inactivate the enzymes, then immediately cooled and filtered under vacuum through paper. The filtrate was weighed to determine the extract yield [45]. The same process was followed for the control sample, except that an equivalent volume of distilled water was used in place of the enzymes.

### 2.3. OLE@LDH_Zn/Al Nanohybrids’ Preparation

First, 50 mL of OLE was filtered under vacuum with 0.45 μm filters. An 8 M aqueous NaOH solution was prepared by dissolving 9.6 g of NaOH pellets in 30 mL of purified water. To obtain final Zn/Al molar ratios of 1:1, 2:1, and 3:1, the appropriate amounts of Zn(NO_3_)_2_·6H_2_O and Al(NO_3_)_3_·9H_2_O were accurately weighed using an analytical balance. The salts were then dissolved in 10 mL of purified water in a 250 mL beaker under stirring. Once fully dissolved, the beaker was placed in a chemical fume hood in an oil bath at 60 °C with continuous stirring. When the solution reached 60 °C, the filtered OLE was added, and stirring continued until the temperature stabilized. The prepared NaOH solution was then added dropwise to the mixture until pH = 7. The reaction mixture was maintained at 60 °C under constant stirring for 24 h. Afterward, the suspension was divided into two centrifuge tubes and centrifuged three times at 3000 rpm (Sorvall Legend X1, Thermo Scientific, Waltham, MA, USA) for 2–4 min. Following the first centrifugation, the pale-yellow supernatant was discarded. A small amount of purified water was added to the remaining sediment, followed by manual stirring and 3 min of ultrasonic treatment. The tubes were then filled with purified water and centrifuged a second time. This washing step was repeated for each centrifugation, although sonication was only applied after the first wash. After the third centrifugation, the now colorless and transparent supernatant was discarded. The remaining sediment was resuspended in a small volume of purified water, manually stirred, and transferred to a glass plate to maximize material recovery. The final product, the OLE@LDH_Zn/Al nanohybrid, was dried overnight at 40 °C.

### 2.4. Phytochemical Analyses of OLE

The determination of HT, lut-7-o-glu, apig-4-o-glu, and oleur in the olive leaf extract was performed following the analytical method proposed by the IOC, in accordance with the conditions described in the IOC/T.20/Doc No 29 method [46]. Separation of components was achieved using a reversed phase Discovery^®^ HS C18 column (250 × 4.6 mm, 5 µm particle size). The mobile phase consisted of 0.2% *v*/*v* aqueous orthophosphoric acid (solvent A) and a methanol/acetonitrile mixture (50:50 *v*/*v*) as solvent B. Chromatographic separation was carried out at ambient temperature with a flow rate of 1.0 mL/min and an injection volume of 20 µL.

The gradient elution was as follows: (i) 0 min: 96% A and 4% B; (ii) 40 min: 50% A and 50% B; (iii) 45 min: 40% A and 60% B; (iv) 60 min: 0% A and 100% B; (v) 70 min: 0 A and 100% B; (vi) 72 min: 96% A and 4% B; and (vii) 82 min: 96% A and 4% B. Chromatograms were recorded at 280 nm.

Quantification of the major compounds, i.e., HT, lut-7-o-glu, apig-4-o-glu, and oleur, was achieved using regression analysis. Calibration curves were constructed for each standard compound.

### 2.5. Instrumental Structural Analysis of OLE@LDH_Zn/Al_x/1 Nanohybrids

The synthesized OLE@LDH_Zn/Al_x/1 nanohybrids were characterized using XRD, FTIR, and HR-SEM. Details of the instrumentation are provided in the Supplementary Material File in detail.

### 2.6. EC_50_ Estimation of OLE@LDH_Zn/Al_x/1 Nanohybrids

For all OLE@LDH_Zn/Al_x/1 nanohybrids, as well as pure freeze-dried OLE, the concentration required to achieve a 50% antioxidant effect (EC_50_) was evaluated by three different assays: ferric reducing antioxidant power (FRAP), 2,2-diphenyl-1-picrylhydrazyl (DPPH) radical scavenging, and 2,2-Azino-bis(3-ethylbenzothiazoline-6-sulfonic acid) diammonium salt (ABTS). For all methods, a SHIMADJU UV/VIS spectrophotometer (UV-1900, Kyoto, Japan) was used. All measurements were done in triplicate.

#### 2.6.1. In Vitro Antioxidant Activity Determination of Pure OLE and Obtained OLE@LDH_Zn/Al_x/1 Nanohybrids via the 2,2-Diphenyl-1-picrylhydrazyl (DPPH) Assay Method

For all synthesized OLE@LDH_Zn/Al_x/1 nanohybrids, as well as pure OLE, the concentration required to achieve 50% antioxidant activity (EC_50_) was calculated following the methodology described recently [47]. To prepare the 2,2-diphenyl-1-picrylhydrazyl (DPPH^•^) free radical standard solution, 0.0212 g of DPPH^•^ was dissolved in 250 mL of ethanol to obtain a 2.16 mM solution. The solution was then vortexed in the dark, and its pH was measured using a Milwaukee MW102-FOOD PRO+ (Milwaukee Electronics Kft. Alsó-Kikötő sor 11 C. H-6726 Szeged, Hungary) 2-in-1 pH and Temperature Meter to confirm neutrality (7.02 ± 0.01). Finally, the solution was stored in the refrigerator at 4 ± 1 °C under dark conditions for stabilization.

To determine the concentration required to achieve 50% antioxidant activity (EC_50_) for all synthesized OLE@LDH_Zn/Al_x/1 nanohybrids and pure OLE, samples of 5, 10, 20, 30, and 40 mg of the nanohybrid granules or 5, 10, 20, 30, and 50 μL of pure OLE were placed in dark vials, with three replicates performed for each. Then, 3 mL of DPPH^•^ methanolic solution and 2 mL of 100 mM acetate buffer (pH 7.10) were added to each vial. After 24 h, the absorbance of the reaction mixture was measured at 517 nm. A blank sample containing 3 mL of DPPH^•^ ethanolic solution and 2 mL of acetate buffer without any nanohybrid or OLE was used as the control. The percentage inhibition of DPPH^•^ was calculated using the following equation:

(1) $$\%\;scavenged\;{DPPH}^{•}\;at\;steady\;state\;=\;\frac{A_{0}^{517}-A_{sample}^{517}}{A_{0}^{517}}\times 100$$

Then, the obtained antioxidant activity values (DPPH^•^) of each OLE@LDH_Zn/Al_x/1 nanohybrid sample were plotted as a function of the volume used (see Supplementary Material File Table S1 and Figure S2), and the obtained linear equations were used to determine the EC_50,DPPH_ values for each sample.

#### 2.6.2. Antioxidant Activity of O OLE@LDH_Zn/Al_x/1 Nanohybrids with the 2,2′-Azino-bis(3-ethylbenzothiazoline-6-sulfonic Acid) Diammonium Salt (ABTS) Assay

First an ABTS stock solution with 7 mM concentration was prepared by adding 900.6 mg of ABTS in 250 mL deionized water under vigorous stirring. For the preparation of 2.45 mM potassium persulfate stock solution, 0.0662 g potassium persulfate was dissolved with a phosphate buffer solution with a pH = 6.8 and diluted in a volumetric flask of 100.0 mL. The preparation of ABTS solution was done by mixing an equal volume of a 7 mM ABTS stock solution with a 2.45 mM potassium persulfate solution. The mixture was then stored in the dark at room temperature for 12–16 h. The ABTS solution was diluted with 10 mmol/L phosphate-buffered saline (PBS, pH 7.4) to an absorbance of 0.70 ± 0.02 at 734 nm. Then, 10, 20, 30, and 40 mg of each OLE@LDH_Zn/Al_x/1 nanohybrid sample was added to 3 mL of the diluted ABTS solution. The absorbance of the mixture was immediately measured at 734 nm after a 1 h incubation in the dark at room temperature. The control sample was prepared without adding the OLE@LDH_Zn/Al_x/1 nanohybrid. The % ABTS scavenging activity was calculated according to the equation:

(2) $$\%\;scavenged\;ABTS\;at\;steady\;state\;=\;\frac{A_{0}^{734}-A_{sample}^{734}}{A_{0}^{734}}\times 100$$

Then, the obtained antioxidant activity values (ABTSs) of each OLE@LDH_Zn/Al_x/1 nanohybrid sample were plotted as a function of the volume used (see Supplementary Material File Table S2 and Figure S3), and the obtained linear equations were used to determine the EC_50,ABTS_ values for each sample.

#### 2.6.3. Antioxidant Activity of O OLE@LDH_Zn/Al_x/1 Nanohybrids with the Ferric Reducing Antioxidant Power (FRAP) Assay

FRAP working solution was prepared freshly each time: 0.3 M acetate buffer (pH = 3.6), 0.01 M TPTZ (2,4,6-tripyridyl-s-triazine) in 0.04 M HCl and 0.02 M FeCl_3_·6H_2_O were mixed at a ratio of 10:1:1 (*v*/*v*/*v*) and kept away from light. Then 10, 20, 30, and 40 mg of each OLE@LDH_Zn/Al_x/1 nanohybrid sample were added to 2.25 mL of FRAP working solution and 0.225 mL of deionized water. The mixture was vortexed and incubated at 37 °C for 30 min away from light. Absorbance was measured at 593 nm. FRAP working solution with deionized water, instead of adding any OLE@LDH_Zn/Al_x/1 nanohybrid, was used as a blank. The % FRAP scavenging activity was calculated according to the following equation:

(3) $$\%\;scavenged\;FRAP\;at\;steady\;state\;=\;\frac{A_{0}^{593}-A_{sample}^{593}}{A_{0}^{593}}\times 100$$

Then, the obtained antioxidant activity values (FRAP) of each OLE@LDH_Zn/Al_x/1 nanohybrid sample were plotted as a function of the volume used (see Supplementary Material File Table S3 and Figure S4), and the obtained linear equations were used to determine the EC_50,FRAP_ values for each sample.

All determinations were carried out in triplicate.

### 2.7. Total Polyphenol Content (TPC) of OLE@LDH_Zn/Al_x/1 Nanohybrids

The TPC of obtained OLE and all the obtained Zn/Al_x/1 nanohybrids was measured by using a SHIMADJU UV/VIS spectrophotometer (UV-1900, Kyoto, Japan) via the following methodology.

TPC of OLE: A 0.2 mL aliquot of OLE was added to a 5 mL volumetric flask containing 2.5 mL of distilled water and 0.25 mL of Folin–Ciocalteu reagent. After 3 min, 0.5 mL of saturated sodium carbonate solution (Na_2_CO_3_, 30% *w*/*v*) was added to create the alkaline environment required for the colorimetric reaction between the Folin–Ciocalteu reagent and phenolic compounds. This reaction was allowed to proceed under alkaline conditions before any pH adjustments were made. The final volume was then adjusted to 5 mL using distilled water for measurements at pH 7, with 1 M citric acid aqueous solution for measurements at pH 3.6, and with 0.1 M HCl aqueous solution for measurements at pH 1. The mixture was incubated in the dark at room temperature for 2 h, after which the absorbance was measured at λ = 760 nm. The results were presented as equivalents of gallic acid (GAE). Each sample was analyzed in triplicate (*n* = 3).

TPC of Zn/Al_x/1 nanohybrids: A total of 10 mg of each nanohybrid was added in 10 mL of ethanol. The mixture was stirred and filtered with 0.45 μm filters to obtain 10 mL of an ethanolic extraction. Then 0.20 mL of the ethanolic extraction was added in a 5 mL volumetric flask, followed by the addition of 2.50 mL of distilled water and 0.25 mL of Folin–Ciocalteu reagent. After 3 min, 0.50 mL of saturated sodium carbonate (Na_2_CO_3_, 30% *w*/*v*) was also added into the mixture. Finally, the solution obtained was brought to 5 mL with distilled water for the measurements at pH = 7, with 1 M citric acid aquatic solution for measurements at pH = 3.6, and with 0.1 M HCl aquatic solution for measurements at pH = 1. This solution was left for 2 h in the dark at room temperature and the absorbance was measured at λ = 760 nm. The results were presented as equivalents of gallic acid (GAE). Each sample was analyzed in triplicate (*n* = 3).

### 2.8. Antibacterial Activity

#### 2.8.1. Antimicrobial Activity of OLE@LDH_Zn/Al_x/1 Nanohybrids

Standard and isolated strains of two bacteria were used to screen antimicrobial activity: one Gram-positive (*Staphylococcus aureus*, ATCC 25923) and one Gram-negative strain (*Escherichia coli*, ATCC 25922). The antimicrobial activity of the OLE@LDH_Zn/Al_x/1 nanohybrids was evaluated using the disk diffusion method in accordance with EUCAST guidelines. The minimum inhibitory concentration (MIC) and minimum bactericidal concentration (MBC) were determined using a resazurin-based 96-well plate microdilution method [48]. The MIC, MBC, and zone of inhibition (ZOI) values represent the mean obtained from three independent experiments.

#### 2.8.2. Disk Diffusion Susceptibility Test

Aseptically, 50 mg of each of the OLE@LDH_Zn/Al_x/1 nanohybrids was weighed and directly applied as a defined circular deposit onto the surface of Mueller–Hinton agar (MHA) plates which had been previously inoculated with the respective test microorganism (108 CFU ml^−1^). A minimum center-to-center distance of 24 mm was maintained between each sample application site to prevent overlapping of the inhibition zones. The inoculated MHA plates were then incubated at 37 °C for 24 h. After the incubation, the diameter of the clear zone of inhibition surrounding each disk was calculated using a calibrated ruler. The actual diameter of inhibition was then calculated by subtracting the initial diameter of the applied sample deposit from this measurement. The test was performed in duplicate for each test microorganism and OLE@LDH_Zn/Al_x/1 nanohybrid concentration.

#### 2.8.3. Resazurin-Based 96-Well Plate Microdilution Method

The MIC determination of OLE@LDH_Zn/Al_x/1 nanohybrid samples was performed following the protocol described by [48]. The analyses were carried out in a microdilution plate with 96 wells (see Figure S4) (sterilized, 300 μL capacity, MicroWell, NUNC, Thermo-FisherScientific, Waltham, MA, USA). The OLE@LDH_Zn/Al_x/1 nanohybrids were dissolved in sterile dH_2_O. Initial solutions of OLE@LDH_Zn/Al_x/1 nanohybrids were prepared at a concentration of 50 mg·ml^−1^, and further serial dilutions were performed in the 96-well plate in Mueller–Hinton broth (MHB). The concentrations tested ranged from 25 to 0.05 mg·mL^−1^. Positive controls (growth control) containing inoculum as well as negative controls with broth (MHB) were prepared. After adding a standardized inoculum of the target bacteria (10^8^ CFU mL^−1^), the microplate was then incubated for 24 h at 37 °C. After incubation, 50 μL of a 0.015% *w*/*v* resazurin solution was added to each well and the plate was incubated for another 2 h. If there is no metabolic activity (inhibition), the resazurin stays blue, and if there is activity (growth), it turns pink/purple. Wells without color change were scored as concentrations above the MIC. Treatments that showed inhibition of microorganisms were then tested for bactericidal activity (MBC) by plating a 10 μL sample onto MHA plates and incubating for 24 h at 37 °C. The lowest treatment concentration that did not show colony formation after incubation in all replicates was considered the MBC.

### 2.9. Statistical Analysis

Descriptive statistics for OLE extraction yield, HΤ content, lut-7-ο-glu content, and oleur content, as well as for EC_50_, TPC, and ZOI parameters, were estimated using SPSS software (version 29.0; IBM Corp., Armonk, NY, USA). The last three properties were also subjected to statistical analysis to investigate the statistically significant differences of their mean values. For such investigation, the statistical test method ANOVA was chosen. A significance level of *p* < 0.05 was adopted for all comparisons.

## 3. Results

### 3.1. HPLC-DAD Analyses of OLE

**Figure 1:**
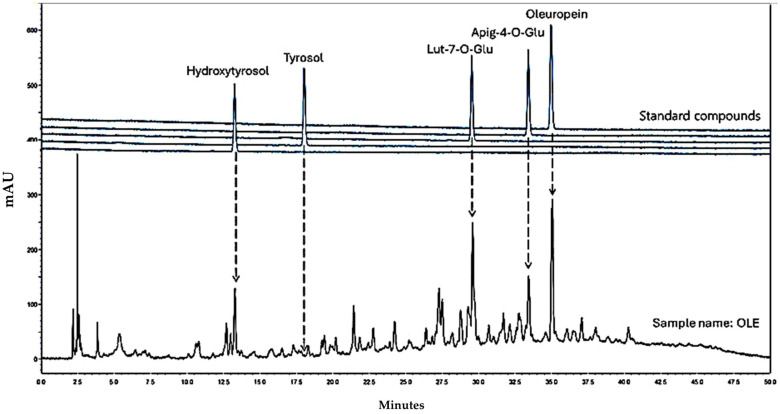
HPLC-DAD chromatogram of pure OLE recorded at 280 nm.

The results of the phytochemical analysis, including the mean values of the total polyphenol yield and the contents of HT, lut-7-ο-glu, apig-4-o-glu, and oleur in the OLE sample, as determined by HPLC-DAD, are summarized in Table 1. These values were calculated using the calibration curves of HT, lut-7-ο-glu, apig-4-o-glu, and oleur (Figure S1), along with the HPLC-DAD chromatogram of pure OLE recorded at 280 nm (Figure 1).

As shown in Table 1, a high extraction yield of 24.0 ± 2.8 mg/L was obtained for the pure OLE. This high extraction yield value is attributed to the optimum combination of viscozyme and pectinolytic enzymes used in the microwave-assisted extraction process [44]. According to the HPLC-DAD analysis results (Table 1), the pure OLE contains 2.19% wt. HT (0.53 mg/L), 2.89% wt. lut-7-ο-glu (0.70 mg/L), 0.75% wt. apig-4-o-glu (0.18 mg/L), and 17.64% wt. oleur (4.24 mg/L). These findings indicate that the extract is rich in total polar phenols, with approximately 25% wt. comprising high value bioactive compounds.

### 3.2. Physicochemical Characterization of OLE@LDH_Zn/Al_x/1 Nanohybrids

#### 3.2.1. XRD Analysis of OLE@LDH_Zn/Al_x/1 Nanohybrids

**Figure 2:**
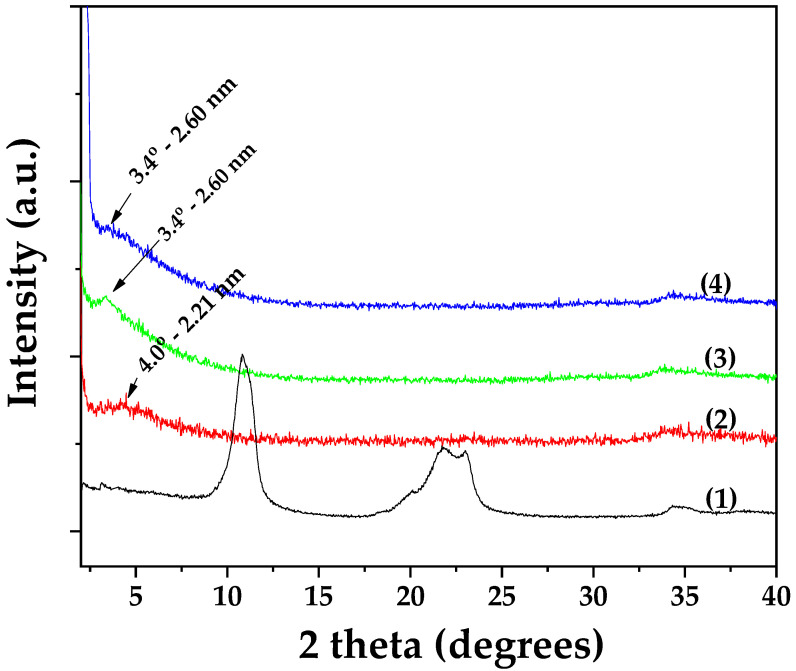
XRD patterns of (1) pure LDH_NaNO_3__Zn/Al, (2) OLE@LDH_Zn/Al_1/1, (3) OLE@LDH_Zn/Al_2/1, and (4) OLE@LDH_Zn/Al_3/1.

Figure 2 presents the XRD patterns of all synthesized OLE@LDH_Zn/Al_x/1 nanohybrids, alongside that of pure LDH_NaNO_3__Zn/Al for comparison. As shown and consistent with previous studies, the characteristic (003) reflection of pure LDH_NaNO_3__Zn/Al (plot line 1) appears at approximately 10°, corresponding to a basal spacing of 0.83 nm [49,50]. In the XRD patterns of the OLE@LDH_Zn/Al_x/1 nanohybrids, a broad peak observed around 5° indicates a shift of the (003) reflection to lower angles. Specifically, the basal spacing increases to 2.21 nm for OLE@LDH_Zn/Al_1/1 (plot line 2) and to 2.6 nm for both OLE@LDH_Zn/Al_2/1 (plot line 3) and OLE@LDH_Zn/Al_3/1 (plot line 4). This variation in basal spacing among the nanohybrids suggests differences in the orientation of the intercalated OLE molecules within the LDH interlayers, rather than differences in the amount of OLE adsorbed [51,52]. Among the three formulations, OLE@LDH_Zn/Al_2/1 exhibits the most intense (003) reflection, indicating superior platelet orientation. Overall, the XRD results confirm the successful intercalation of OLE molecules into the interlayer space of the LDH structure.

#### 3.2.2. FTIR of OLE@LDH_Zn/Al_x/1 Nanohybrids

**Figure 3:**
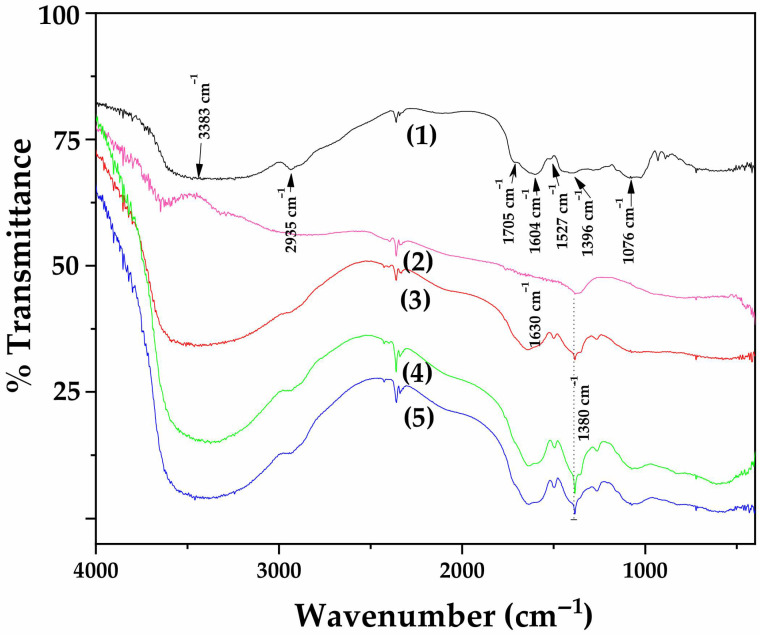
FTIR plots of (1) pure OLE, (2) pure LDH_NaNO_3__Zn/Al, (3) OLE@LDH_Zn/Al_1/1, (4) OLE@LDH_Zn/Al_2/1, and (5) OLE@LDH_Zn/Al_3/1.

Figure 3 displays the FTIR spectra of all synthesized OLE@LDH_Zn/Al_x/1 nanohybrids, along with those of pure LDH_NaNO_3__Zn/Al and pure OLE for comparison.

The FTIR spectrum of pure OLE (plot line 1) displays several absorption peaks, reflecting its complex chemical composition. The peak at 3383 cm^−1^ can be attributed to the N–H stretching vibration of amino groups and also indicates the presence of a bonded hydroxyl (–OH) group [53]. The absorption peak at 2935 cm^−1^ corresponds to –CH stretching vibrations of both –CH_3_ and –CH_2_ functional groups [53,54,55,56]. The shoulder peak at 1705 cm^−1^ is assigned to the C=O stretching vibration of carboxylic acids. These two prominent bands at 3383 and 1705 cm^−1^ are characteristic of the O–H and C=O stretching modes, potentially originating from compounds such as οleur, apig-4-o-glu, and/or lut-7-ο-glu [53,54,57,58]. The peak at 1604 cm^−1^ lies within the fingerprint region and is associated with the presence of CO, C–O, and O–H functional groups found in OLE [4]. Specifically, it can be attributed to C–O stretching in carboxyl groups coupled with the amide I linkage. The band at 1527 cm^−1^, characteristic of amide II, arises from N–H stretching modes in the amide linkage [59]. The peak at 1396 cm^−1^ is attributed to methylene scissoring vibrations associated with protein content. A strong band at 1076 cm^−1^ is assigned to C–N stretching vibrations of aliphatic amines or C–OH vibrations in proteins derived from olive leaves [53,54,57,58,60].

In the FTIR spectra of all synthesized LDHs (plot lines 2, 3, 4, and 5), characteristic bands of hydrotalcite-like compounds are revealed. The broad and intense band centered at 3620 cm^−1^ is attributed to the stretching of OH groups and adsorbed H_2_O molecules [61]. The region between 603 and 430 cm^−1^ corresponds to Al–O and Zn–O vibrational modes [61]. Additionally, a weak band at 1630 cm^−1^ is attributed to the bending vibration of interlayer water molecules [61]. The FTIR spectrum of LDH_NaNO_3__Zn/Al reveals a strong peak near 1380 cm^−1^, corresponding to the antisymmetric stretching mode (v_3_) of nitrate anions present in the LDH structure [61]. Comparing the FTIR spectra of pure LDH_NaNO_3__Zn/Al with those of all OLE@LDH_Zn/Al_x/1 nanohybrids reveals a significant difference: a large, broad peak around 3500 cm^−1^ appears in all nanohybrid spectra. This peak results from overlapping hydroxyl group vibrations from both the LDH and OLE, indicating strong interactions between the intercalated hydroxyl groups of OLE and the internal hydroxyl groups of the LDH platelets.

#### 3.2.3. HR-SEM Analysis of OLE@LDH_Zn/Al_x/1 Nanohybrids

**Figure 4:**
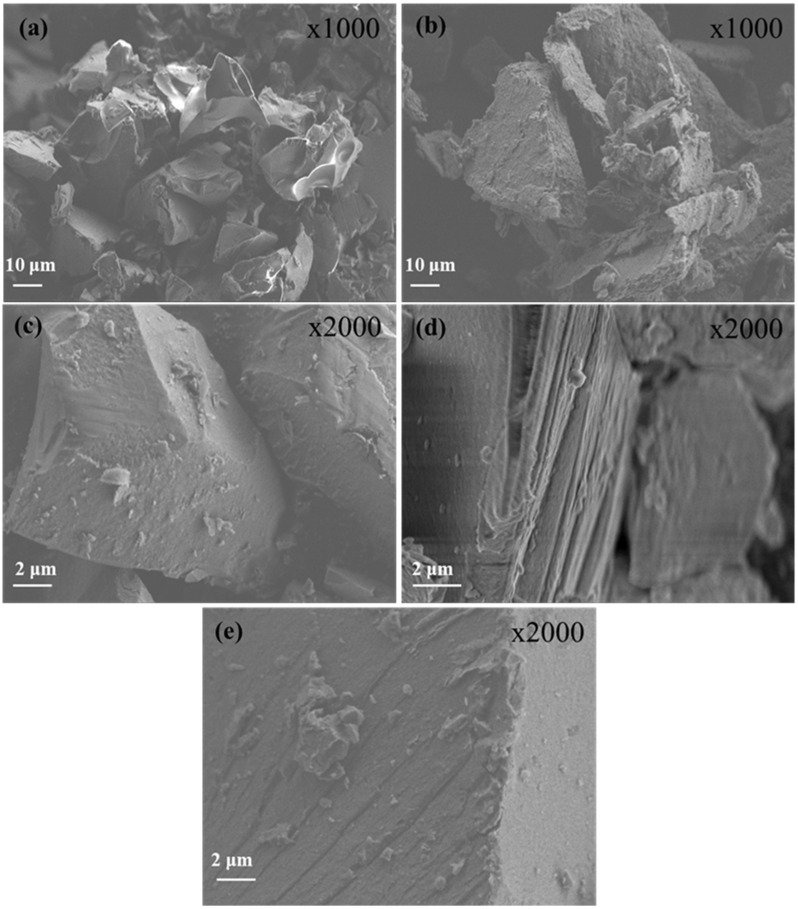
SEM images of (**a**) pure freeze-dried OLE, (**b**) pure LDH_NaNO_3__Zn/Al, (**c**) OLE@LDH_Zn/Al_1/1, (**d**) OLE@LDH_Zn/Al_2/1, and (**e**) OLE@LDH_Zn/Al_3/1.

The morphological characteristics of pure OLE and LDH along with the corresponding nanohybrids are presented in Figure 4. Representative images of pure freeze-dried OLE and LDH are shown in Figure 4a and Figure 4b, respectively. The pure freeze-dried OLE structure has a sponge-like structure with large holes, which indicate the evaporated water molecules. The pure LDH structure reveals an irregular disk-like or flake-like morphology with obvious lamellar structure, which is a typical characteristic of LDH nanostructures [62,63]. Larger platelets or clusters of LDH are also observed, indicating their tendency to agglomerate or overlap due to interlayer forces and particle–particle interactions [47,63]. The SEM images of LDH-modified nanohybrids (see images in Figure 4c–e) reveal notable morphological changes compared to the unmodified nanostructures. In all OLE@LDH_Zn/Al_x/1 nanohybrids, large sandwich-like particles are observed, suggesting successful OLE intercalation and particle agglomeration. Additionally, irregular nanostructures or particles appear on the surface of the LDH layers, which can be attributed to the OLE nanostructure. Among the nanohybrids, the sandwich-like morphology is most pronounced in OLE@LDH_Zn/Al_2/1. This observation is consistent with the XRD results, which indicate that the most favorable platelet orientation occurs in the OLE@LDH_Zn/Al_2/1 nanohybrid.

### 3.3. Antioxidant Activity of OLE@LDH_Zn/Al_x/1 Nanohybrids

#### 3.3.1. EC_50_ Estimation

The antioxidant activity of all obtained OLE@LDH_Zn/Al_x/1 nanohybrids as well as pure OLE was evaluated via the DPPH, ABTS, and FRAP assay methods and the determination of the effective concentration for 50% antioxidant activity (EC_50_). Calculated mean values of EC_50,DPPH_, EC_50,ABTS_, and EC_50,FRAP_ are listed in Table 2 for comparison.

As shown in Table 2, the EC_50_ values measured via different assay methods follow the same trend for all OLE@LDH_Zn/Al_x/1 nanohybrids. Thus, OLE@LDH_Zn/Al_1/1 exhibited the lowest EC_50,DPPH_, EC_50,ABTS_, and EC_50,FRAP_ values, while OLE@LDH_Zn/Al_3/1 exhibited the highest EC_50,DPPH_, EC_50,ABTS_, and EC_50,FRAP_ values. Statistical hypothesis analysis showed that in all cases of EC_50_ parameters, the mean value of OLE, OLE@LDH_Zn/Al_1/1, and OLE@LDH_Zn/Al_2/1 were statistically equal, while the only statistically different value occurred for the sample OLE@LDH_Zn/Al_3/1. An exception occurs in the case of EC_50,FRAP_ measurements, where OLE and OLE@LDH_Zn/Al_1/1 samples exhibited statistically equal mean values, while the OLE@LDH_Zn/Al_2/1 and OLE@LDH_Zn/Al_3/1 samples exhibited statistically different mean values. This result indicates that as the Zn/Al molar ratio increases, the EC_50,DPPH_, EC_50,ABTS_, and EC_50,FRAP_ values also increase, reflecting a corresponding decrease in antioxidant activity. This trend can be attributed to the increasing positive charge of the LDH layers as the Zn content decreases. More positively charged LDH sheets can intercalate a greater amount of negatively charged polar phenols from the OLE, which explains the superior antioxidant performance of the OLE@LDH_Zn/Al_1/1 nanohybrid [64,65].

**Figure 5:**
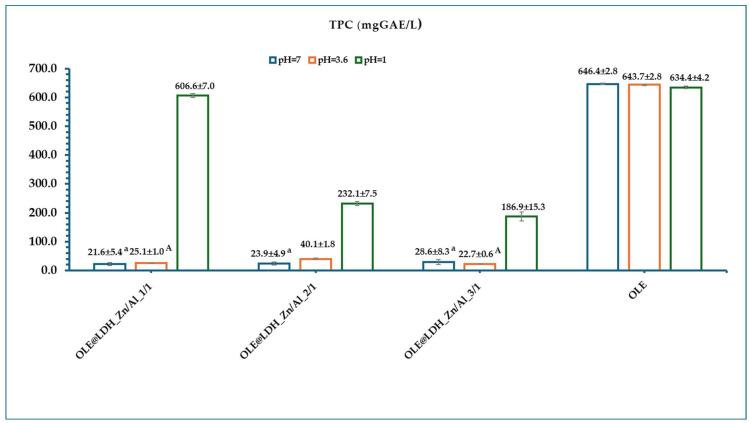
Calculated mean TPC values of all obtained OLE@LDH_Zn/Al_x/1 nanohybrids in pH = 7, 3.6, and 1, as well as pure OLE. Different letters (a, A) in each bar indicate statistically significant differences at a confidence level of *p* < 0.05. See also Table S7 in the Supplementary Material File.

The Zn/Al molar ratio is well known to influence the photocatalytic, optical, and dielectric properties of Zn–Al LDHs [66]. Herein, it is reported for the first time that this ratio also plays a critical role in determining the antioxidant capacity of OLE@LDH_Zn/Al_x/1 nanohybrids. Furthermore, as shown in Figure 5, the antioxidant capacity of the OLE@LDH_Zn/Al_1/1 nanohybrid exceeds that of the pure freeze-dried OLE, suggesting a possible synergistic effect between the antioxidant activity of the nanoencapsulated polar phenols and that of the Zn/Al LDH matrix.

#### 3.3.2. Total Phenolic Content (TPC) Estimation

Figure 5 presents the calculated mean TPC values of all OLE@LDH_Zn/Al_x/1 nanohybrids at pH = 7, 3.6, and 1. As shown in Figure 5, pure OLE exhibits high mean TPC values of 646.4 ± 2.8, 643.7 ± 2.8, and 634.4 ± 4.2 mg GAE/L at pH = 7, 3.6, and 1, respectively, indicating that polar phenols remain stable across a wide pH range. In contrast, all OLE@LDH_Zn/Al_x/1 nanohybrids show very low TPC values at pH = 7 and 3.6, suggesting that the polar phenols remain encapsulated within the LDH interlayers and are not released into the surrounding medium. However, at pH = 1, a significant increase in TPC is observed for all nanohybrids, with mean values of 606.6 ± 7.0 mg GAE/L for OLE@LDH_Zn/Al_1/1, 232.1 ± 7.5 mg GAE/L for OLE@LDH_Zn/Al_2/1, and 186.9 ± 15.3 mg GAE/L for OLE@LDH_Zn/Al_3/1. This confirms that under acidic conditions, mimicking the stomach environment (pH = 1.2–1.8), the LDH structure disintegrates, releasing the encapsulated polar phenols. These findings demonstrate that the LDH nanocarriers effectively protect the OLE polar phenols at neutral and mildly acidic pHs, while enabling their controlled release in gastric conditions, supporting their potential use in nutritional and biomedical applications [67,68].

Moreover, the TPC trends align with the antioxidant activity results: both EC_50_ and TPC values follow the same pattern, with OLE@LDH_Zn/Al_1/1 exhibiting the highest values. This further supports that the 1:1 Zn/Al molar ratio yields the most positively charged LDH layers, allowing greater polyphenol loading and thus superior antioxidant performance.

### 3.4. Antibacterial Activity of OLE@LDH_Zn/Al_x/1 Nanohybrids

The antibacterial activity results of all OLE@LDH_Zn/Al_x/1 nanohybrids against *E. coli* and *S. aureus*, assessed by the ZOI, MIC, and MBC, are summarized in Table 3 for comparison. As shown, all nanohybrids demonstrated significant antibacterial activity against both bacterial strains across all methods. These findings are consistent with previous studies in which OLE encapsulated in maltodextrin-based and maltodextrin/casein-based matrices exhibited notable antibacterial activity against both gram-positive and gram-negative bacteria [69,70]. Among the three assessment methods, MIC and MBC are considered more reliable for drawing comparative conclusions on antibacterial efficacy [70,71,72,73,74]. Based on these metrics, the OLE@LDH_Zn/Al_1/1 nanohybrid exhibited the strongest antibacterial activity against both *E. coli* and *S. aureus*. This result aligns with its highest TPC and lowest EC_50_ values, further validating the enhanced bioactivity of this formulation and supporting the overall findings of the study.

## 4. Discussion

In this study, olive leaf valorization was achieved through an environmentally friendly enzyme-assisted extraction method, yielding an aqueous, polyphenol-rich olive leaf extract containing HT (0.53 mg/L), lut-7-ο-glu (0.70 mg/L), apig-4-ο-glu (0.18 mg/L), and oleur (4.24 mg/L). Olive leaves, typically considered agricultural and industrial waste, possess significant potential for economic and medicinal applications [68,69,73,74]. While previous studies have explored the extraction of OLE using various solvent systems, including ethanol or ethanol/water mixtures with acetic acid, the enzyme-assisted aqueous extraction used here offers a simpler, greener, and more cost-effective alternative [69,74]. For instance, Tarchi et al. [69] produced an oleuropein-rich aqueous extract with a TPC of 395.45 ± 8.21 mg GAE/g via autoclave, a method that, although effective, is more complex and expensive than the one employed in this work. Following extraction, the OLE was nano-encapsulated in LDHs by varying the Zn^2+^/Al^3+^ molar ratio to 1:1, 2:1, and 3:1. Previous encapsulation efforts utilized organic matrices. For example, Medfai et al. [70] encapsulated ethanolic OLE using spray-drying with maltodextrin-based systems. Oliveira et al. [74] used gelatin/tragacanth gum blends, and Tarchi et al. [69] employed maltodextrin and sodium caseinate to enhance compound stability and bioavailability. This work, however, is the first to report the successful nanoencapsulation of OLE in inorganic LDH nanocarriers.

The obtained OLE@LDH_Zn/Al_1/1, OLE@LDH_Zn/Al_2/1, and OLE@LDH_Zn/Al_3/1 nanohybrids were physiochemically characterized via XRD, FTIR, and SEM analysis, which clearly showed the incorporation of OLE inside the interlayer space of LDHs in all cases to obtain intercalated nanocomposite structures. Both antioxidant and antibacterial activity tests revealed that the OLE@LDH_Zn/Al_1/1 nanohybrid has the lowest EC_50,DPPH_, EC_50,ABTS_, and EC_50,FRAP_ values (27.88 ± 1.82, 25.70 ± 0.76, and 39.42 ± 2.16 mg/mL), and the highest MIC (3.12–1.56 mg/mL) and MBC (12.5–6.25 mg/mL) values against *E. coli* and *S. aureus*, correspondingly. The antioxidant and antibacterial results align, with the highest TPC (606.6 ± 7.0 mgGAE/L) value also obtained for the OLE@LDH_Zn/Al_1/1 nanohybrid. The highest antioxidant and antibacterial activity of the OLE@LDH_Zn/Al_1/1 nanohybrid is in line with its highest TPC capacity, exhibited due to its highest amount of positive sheets of LDH, which can intercalate a greater amount of negatively charged OLE.

Thus, this study not only reports, for the first time, the preparation and characterization of OLE@LDH_Zn/Al_x/1 nanohybrids, but also identifies the 1:1 Zn^2+^/Al^3+^ molar ratio as the most effective formulation for OLE encapsulation. Moreover, TPC measurements at various pH levels clearly demonstrated that the encapsulated OLE is well-protected at neutral (pH = 7) and mildly acidic (pH = 3.6) conditions, and is effectively released under highly acidic conditions (pH = 1), mimicking the gastric environment.

Overall, this work demonstrates that (i) LDHs are highly promising nanocarriers for the encapsulation and controlled release of OLE under stomach-like conditions, and (ii) the OLE@LDH_Zn/Al_1/1 nanohybrid exhibits strong potential for future applications in nutrition, food preservation, and biomedicine.

## 5. Conclusions

This work introduces an efficient, green valorization strategy for olive leaves via enzymatic-assisted extraction, yielding a polyphenol-rich OLE containing HT, lut-7-ο-glu, apig-4-ο-glu, and oleur. For the first time, this extract was successfully nanoencapsulated into LDHs at varying Zn^2+^/Al^3+^ molar ratios. Comprehensive structural and functional characterizations confirmed the formation of intercalated nanohybrids, with the 1:1 Zn/Al ratio (OLE@LDH_Zn/Al_1/1) demonstrating superior performance. This hybrid exhibited the highest total polyphenol content, the strongest antioxidant activity, and the most potent antibacterial effect among the tested formulations. Furthermore, pH-responsive release studies revealed that OLE polar phenols are effectively retained within the LDH interlayers under neutral and mildly acidic conditions, and are selectively released under acidic conditions mimicking gastric pH. These findings underscore the potential of LDH-based nanohybrids as effective delivery systems for plant-derived polar phenols, with promising applications in food preservation, nutrition, and biomedicine. Future work will include benchmarking against other nanocarrier systems, encapsulating a broader range of bioactive compounds, and assessing performance in eukaryotic cell models to further explore their practical applicability.

## Figures and Tables

**Table 1:** Phytochemical analysis of pure OLE.

Sample Name	Extraction Yield (mg/L)	HT (mg/L)	lut-7-ο-glu (mg/L)	apig-4-o-glu (mg/L)	oleur (mg/L)
**OLE**	24.00 ± 0.02	0.53 ± 0.02	0.70 ± 0.02	0.18 ± 0.01	4.24 ± 0.03

**Table 2:** Calculated mean and standard deviation values of EC_50,DPPH_, EC_50,ABTS_, and EC_50,FRAP_, of all OLE@LDH_Zn/Al_x/1 nanohybrids as well as pure freeze-dried OLE.

Sample	EC_50,DPPH_ (mg/mL)	EC_50,ABTS_ (mg/mL)	EC_50,FRAP_ (mg/mL)
OLE@LDH_Zn/Al_1/1	27.88 ± 1.82 ^a^	25.70 ± 0.76 ^a^	39.42 ± 2.16 ^a^
OLE@LDH_Zn/Al_2/1	35.62 ± 4.80 ^a^	35.33 ± 3.24 ^a^	52.71 ± 5.01
OLE@LDH_Zn/Al_3/1	69.07 ± 10.50	67.82 ± 8.59	98.68 ± 4.96
OLE	30.56 ± 0.48 ^a^	28.25 ± 0.92 ^a^	41.96 ± 1.37 ^a^

^a^: Different letters in the same column indicate significant differences between mean values (Tukey HSD, *p* < 0.05). See also Tables S4–S7 in the Supplementary Material File.

**Table 3:** Comparison of antimicrobial activities from OLE@LDH_Zn/Al_x/1 against *E. coli* and *S. aureus* via the ZOI, MIC, and MBC methods.

Bacteria	Sample	MIC * (mg/mL)	MBC * (mg/mL)	ZOI * (mm)
*E. coli*				
	1/1 ^#^	3.12	12.5	5 ± 1 ^a^
	2/1 ^#^	3.12	6.25	6 ± 1 ^a^
	3/1 ^#^	3.12	6.25	6 ± 1 ^a^
*S. aureus*				
	1/1 ^#^	1.56	6.25	3 ± 1 ^b^
	2/1 ^#^	0.78	6.25	4 ± 1 ^b^
	3/1 ^#^	1.56	3.12	4 ± 1 ^b^

^#^ 1/1: OLE@LDH_Zn/Al_1/1, 2/1: OLE@LDH_Zn/Al_2/1, 3/1: OLE@LDH_Zn/Al_3/1. ^a,b^: Different letters in the same column indicate significant differences between mean values (Tukey HSD, *p* < 0.05). See also Tables S4–S7 in the Supplementary Material File. * MIC: minimum inhibitory concentration; MBC: minimum bactericidal concentration; ZOI: zone of inhibition.

## Data Availability

The datasets generated for this study are available on request to the corresponding author.

## Supplementary Materials

The following supporting information can be downloaded at https://www.mdpi.com/article/10.3390/antiox14081010/s1. Figure S1: Calibration curves obtained for (a) HT, (b) lut-7-ο-glu, (c) apig-4-o-glu, and (d) oleur. Figure S2: Linear plots used for the calculation of average values of EC50,DPPH. Figure S3: Linear plots used for the calculation of average values of EC50,ABTS. Figure S4: Linear plots used for the calculation of average values of EC50,FRAP. Figure S5: Results from three independent replicates for the determination of the minimum bactericidal concentrations (MBCs) of all OLE@LDH_Zn/Al_x/1 nanohybrids against E. coli and S. aureus. Representative images from the resazurin-based 96-well plate microdilution method used for MBC determination are also shown. Table S1: Experimental data used for the calculation of obtained average EC50,DPPH values. Table S2: Experimental data used for the calculation of obtained average EC50,ABTS values. Table S3: Experimental data used for the calculation of obtained average EC50,FRAP values. Table S4: Statistical analysis results of EC50,DPPH values. Table S5: Statistical analysis results of EC50,ABTS values. Table S6: Statistical analysis results of EC50,FRAP values. Table S7: Statistical analysis results of TPC values.
