# Supplementary material for: Antioxidant Nanohybrid Materials Derived via Olive Leaf Extract Incorporation in Layered Double Hydroxide: Preparation, Characterization, and Evaluation for Applications
Source: Antioxidants (Basel). 2025 Aug 18;14(8):1010. doi: 10.3390/antiox14081010 (PMC12382658; doi:10.3390/antiox14081010)
Supplement: Supplementary file 1 [file antioxidants-14-01010-s001.zip › antioxidants-3773010-supplementary.pdf]

Supplementary material for

Antioxidant nanohybrid materials derived via olive leaves extract incorporation in layered double hydroxide: Preparation, characterization, and evaluation for applications

Achilleas Kechagias[1], Areti A. Leontiou[1], Alexios Vardakas[1,2], Panagiotis Stathopoulos[3], Maria Xenaki[4], Panayiota Stathopoulou[5], Charalampos Proestos[6], Emmanuel P. Giannelis[7], Nikolaos Chalmpes[7,*], Constantinos E. Salmas[7,8*], Aris E. Giannakas[1,*]

[1] Department of Food Science and Technology, University of Patras, 30100 Agrinio, Greece; up1110842@upatras.gr (A.K.), aleontiu@upatras.gr (A.L.); alexvard@upatras.gr (A.V.);

[2] GAEA Products S.M. S.A., 1st km Agriniou-Karpenissiou National Rd., GR-30100 Agrinio, Greece

[3] Division of Pharmacognosy and Natural Products Chemistry, Department of Pharmacy, National and Kapodistrian University of Athens, Athens, Greece (P.St.); stathopan@pharm.uoa.gr

[4] PharmaGnose S.A., Papathanasiou 24, 34100 Chalkida, Greece (M.X.); maxenaki@pharm.uoa.gr

[5] Department of Sustainable Agriculture, University of Patras, 30100 Agrinio, Greece; panstath@upatras.gr

[6] Laboratory of Food Chemistry, Department of Chemistry, National and Kapodistrian University of Athens Zografou, 15771 Athens, Greece; harpro@chem.uoa.gr (C.P.)

[7] Department of Materials Science and Engineering, Cornell University, Ithaca, New York 14850, United States; epg2@cornell.edu (E.P.G.)

[8] Department of Material Science and Engineering, University of Ioannina, 45110 Ioannina, Greece;

* Correspondence: agiannakas@upatras.gr (A.E.G.); ksalmas@uoi.gr (C.E.S.), nc427@cornell.edu (N.C.),

Materials and Methods

Quantitative Analysis of Hydroxytyrosol, Luteolin-7-O-glucoside, Apigenin-4-O-glucoside and Oleuropein of OLE with HPLC-DAD method

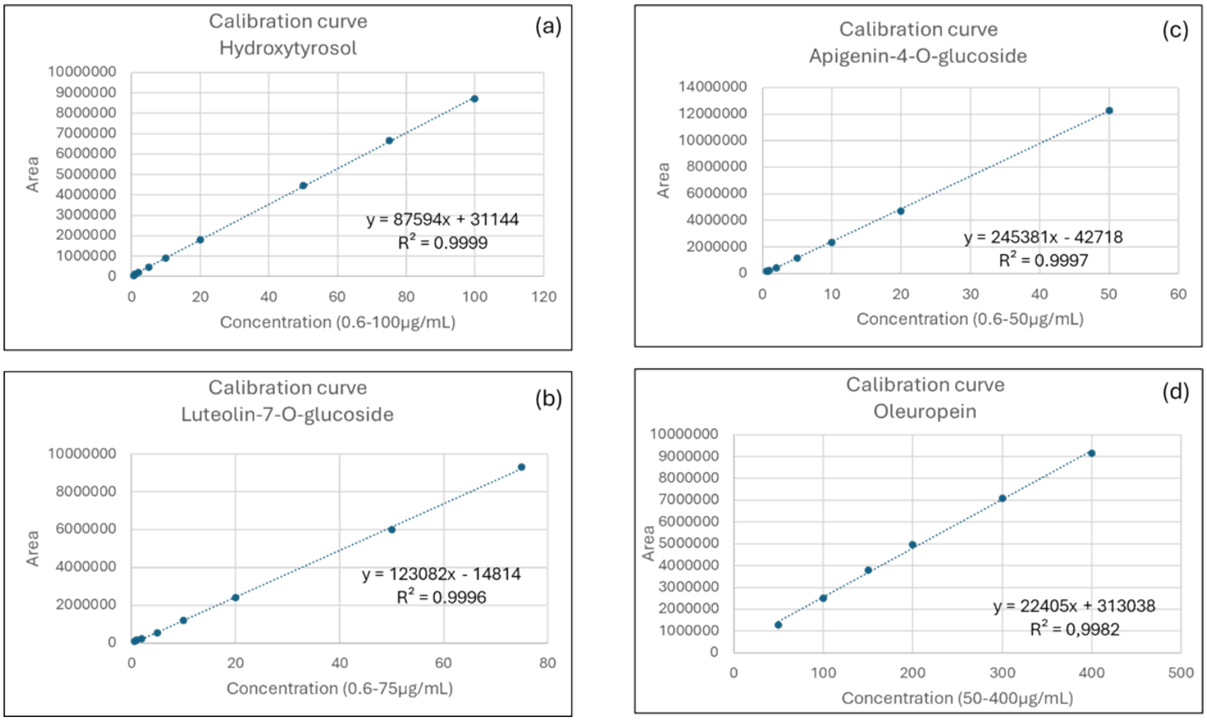

Figure S1. Calibration curves obtained for (a) HT, (b) lut-7-o-glu, (c) apig-4-o-glu, and (d) oleur.

Physicochemical characterization of OLE@LDH_Zn/Al_x/1 nanohybrids

X-Ray Diffraction (XRD) studies

The synthesized OLE@LDH_Zn/Al_x/1 nanohybrids were characterized by X-ray diffraction (XRD) using a Bruker D8 Advance diffractometer (Bruker Analytical Instruments S.A., Athens, Greece) equipped with a LINXEYE XE high-resolution energy-dispersive detector. The diffractometer was thermostated at 20 °C, and the beam monochromator was operated at 40 kV and 40 mA. CuKα radiation (λ = 1.541874 Å) was used in 1-D mode. Scanning parameters were set as follows: 2θ range of 0.5–40°, step size of 0.03°, PSD of 0.764, counting time of 1022 s, and slit width of 0.6 mm. Each sample was mounted on a glass sample holder and carefully aligned to ensure the surface was level with the instrument's reference plane.

Fourier Transform Infrared Spectroscopy (FTIR) studies

FTIR spectra of pure OLE and all synthesized OLE@LDH_Zn/Al_x/1 nanohybrids were recorded using a JASCO FT/IR-6000 Fourier-transform spectrometer (JASCO, Interlab S.A., Athens, Greece). Measurements were performed using the KBr pellet technique, with OLE samples comprising 0.5–1.0 wt% of the pellet. Spectra were recorded over the wavenumber range of 4000–400 cm^{-1} with a resolution of 4 cm^{-1}, and 64 scans were averaged to minimize noise.

Scanning Electron Microscopy (SEM) studies

High Resolution Scanning electron microscopy (HR-SEM) images of pure OLE and all obtained OLE@LDH_Zn/Al_x/1 nanohybrids acquired using a Zeiss Gemini 500 SEM at a low accelerating voltage of 3 kV to reduce the excitation volume and enhance resolution.

Table S1. Experimental data used for the calculation of obtained average $EC_{50,DPPH}$ values. Obtained % absorbance (%AA) values (in triplicate), calculated $EC_{50,DPPH}$ values (in triplicate) and calculated mean $EC_{50,DPPH}$ value for all tested samples.

**A0**		**1.627**	**1.616**	**1.621**	**1.621333333**						
		1	2	3		**%AA_1**	**%AA_2**	**%AA_3**	**average %AA**	**EC50**	**EC50 stdev**
**LDH-OLE_Zn-Al_1-1**	10	1.519	1.516	1.5	1.511666667	6.63798402	6.822372465	7.805777505	6.763980263	**27.61019451**	**1.352162922**
	20	1.409	1.41	1.412	1.410333333	13.39889367	13.33743085	13.21450522	13.01398026	27.05407021	
	30	1.368	1.34	1.308	1.338666667	15.91886908	17.6398279	19.60663798	17.43421053	29.1517395	
	40	1.2	1.239	1.205	1.2195	26.244622	23.84757222	25.93730793	24.78412829	26.62477381	
		1	2	3							
**LDH-OLE_Zn-Al_2-1**	10	1.511	1.532	1.544	1.529	7.12968654	5.838967425	5.101413645	5.694901316	**37.61313672**	**4.87256229**
	20	1.409	1.439	1.427	1.425	13.39889367	11.55500922	12.292563	12.109375	41.05855856	
	30	1.377	1.391	1.392	1.386666667	15.36570375	14.50522434	14.44376152	14.47368421	95.45786678/3	
	40	1.321	1.312	1.297	1.31	18.80762139	19.36078672	20.28272895	19.20230263	34.16771488	
		1	2	3							
**LDH-OLE_Zn-Al_3-1**	10	1.542	1.571	1.572	1.561666667	5.224339275	3.44191764	3.380454825	3.680098684	**69.06678556**	**10.49671755**
	20	1.537	1.514	1.525	1.525333333	5.53165335	6.945298095	6.26920713	5.921052632	57.92339916	
	30	1.478	1.536	1.476	1.496666667	9.157959435	5.593116165	9.280885065	7.689144737	70.50934034	
	40	1.401	1.444	1.478	1.441	13.89059619	11.24769514	9.157959435	11.12253289	78.76761717	
		1	2	3							
**A0**		1.647	1.629	1.636	1.637333333						
**OLE**	10	1.561	1.556	1.55	1.555666667	4.05654579	4.363859865	4.732636755	4.987785016	**30.55818336**	**0.48040884**
	20	1.452	1.478	1.467	1.465666667	10.75599262	9.157959435	9.8340504	10.48452769	30.00818139	
	30	1.379	1.387	1.383	1.383	15.24277812	14.7510756	14.99692686	15.53338762	30.77060611	
	40	1.276	1.283	1.253	1.270666667	21.57344806	21.14320836	22.98709281	22.39413681	30.89576256	

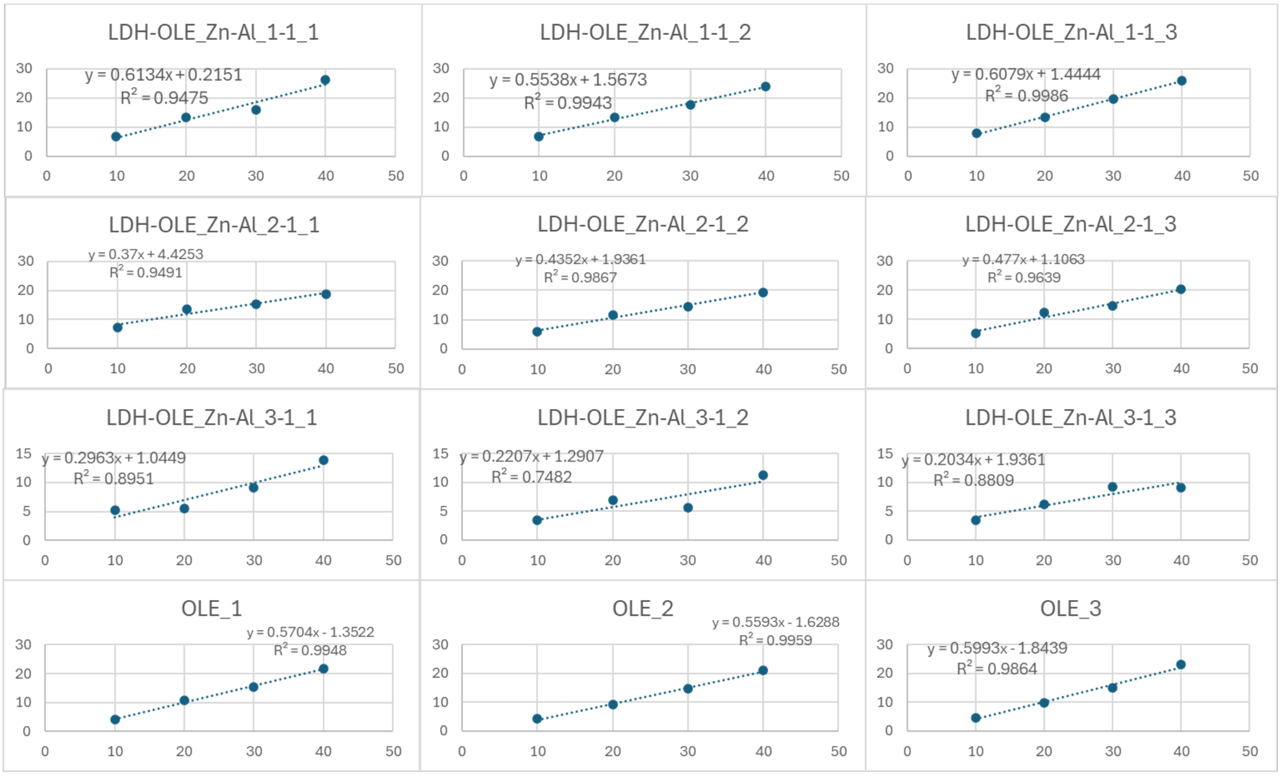

Figure S2. Calculated from the Table S1 linear plots (in triplicate) which are used for the calculation of average values of $EC_{50,DPPH}$ for each tested sample.

Table S2. Experimental data used for the calculation of obtained average $EC_{50,ABTS}$ values. Obtained % absorbance (%AA) values (in triplicate), calculated $EC_{50,ABTS}$ values (in triplicate) and calculated mean $EC_{50,ABTS}$ value for all tested samples.

**A0**		**1.787**	**1.616**	**1.621**	**1.674666667**						
		1	2	3		**%AA_1**	**%AA_2**	**%AA_3**	**average %AA**	**EC50 ABTS**	**EC50 ABTS stdev**
**LDH-OLE_Zn-Al_1-1**	10	1.62533	1.62212	1.605	1.617483333	9.047006156	9.226636821	10.18466704	3.414609873	**25.69611551**	**0.759376042**
	20	1.50763	1.5087	1.51084	1.509056667	15.63346391	15.57358702	15.45383324	9.889132166	26.33311022	
	30	1.46376	1.4338	1.39956	1.432373333	18.08841634	19.76496922	21.68102966	14.46815287	24.85576542	
	40	1.284	1.32573	1.28935	1.304865	28.14773363	25.81253497	27.84834919	22.08210589	25.8994709	
**LDH-OLE_Zn-Al_2-1**	10	1.58655	1.6086	1.6212	1.60545	11.21712367	9.983212087	9.278119754	4.133160828	**35.53321856**	**3.240009023**
	20	1.47945	1.51095	1.49835	1.49625	17.21040851	15.44767767	16.15277001	10.65386146	38.98595797	
	30	1.44585	1.46055	1.4616	1.456	19.09065473	18.26804701	18.20928931	13.05732484	35.05448718	
	40	1.38705	1.3776	1.36185	1.3755	22.38108562	22.90990487	23.79127029	17.86425159	32.55921053	
**LDH-OLE_Zn-Al_3-1**	10	1.58826	1.604143	1.61916	1.6038542	11.12143257	10.23264689	9.39227756	4.228451433	**67.82264936**	**8.589779152**
	20	1.58311	1.598941	1.57075	1.584267033	11.40962507	10.52372132	12.10128707	5.398067277	57.92339916	
	30	1.52234	1.537563	1.52028	1.5267278	14.81029659	13.95839955	14.92557359	8.83392914	73.30887491	
	40	1.44303	1.45746	1.52234	1.474276767	19.24846111	18.44094572	14.81029659	11.9659574	72.235674	
**A0**		1.71288	1.69416	1.70144	1.702826667						
**OLE**	10	1.62344	1.61824	1.612	1.617893333	9.152770006	9.443760492	9.792949077	4.987785016	**28.24957471**	**0.915064926**
	20	1.51008	1.53712	1.52568	1.524293333	15.49636262	13.98321209	14.62339116	10.48452769	28.3706721	
	30	1.43416	1.44248	1.43832	1.43832	19.74482373	19.27923895	19.51203134	15.53338762	29.09806143	
	40	1.32704	1.33432	1.30312	1.321493333	25.73922776	25.33184107	27.077784	22.39413681	27.2799906	

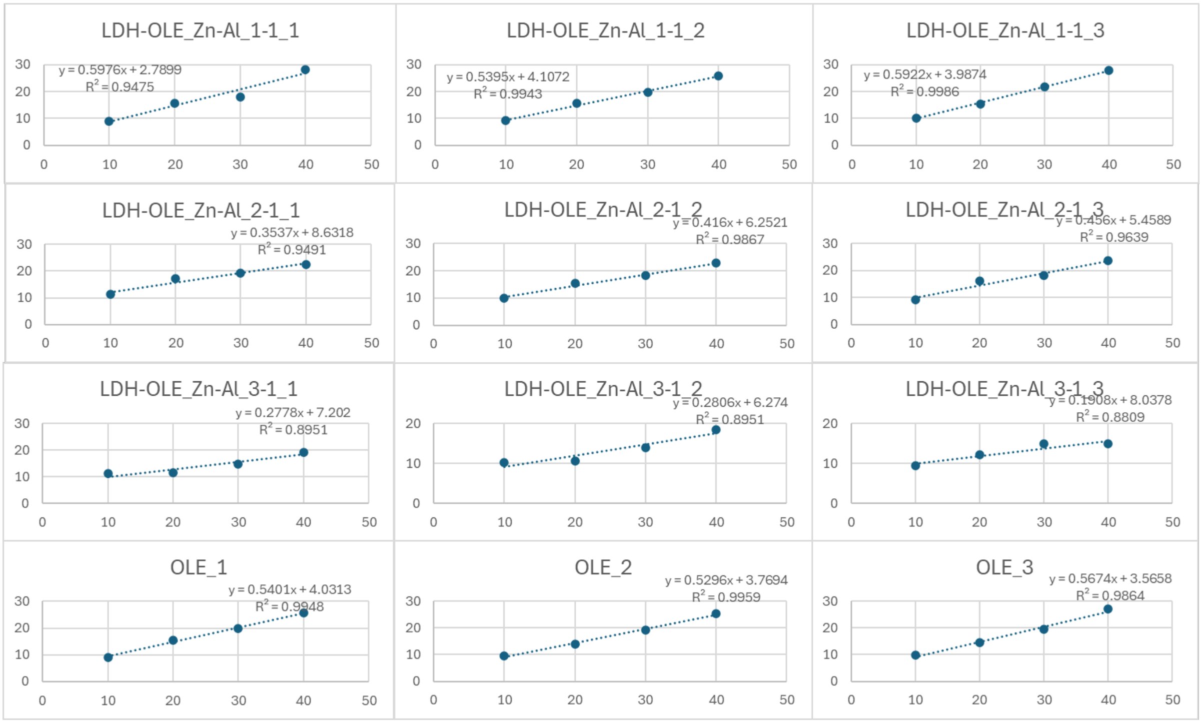

Figure S3. Calculated from the Table S2 linear plots (in triplicate) which are used for the calculation of average values of $EC_{50,ABTS}$ for each tested sample.

Table S3. Experimental data used for the calculation of obtained average $EC_{50,FRAP}$ values. Obtained % absorbance (%AA) values (in triplicate), calculated $EC_{50,FRAP}$ values (in triplicate) and calculated mean $EC_{50,FRAP}$ value for all tested samples.

**A0**		**1.587**	**1.616**	**1.621**	**1.608**						
		1	2	3		**%AA_1**	**%AA_2**	**%AA_3**	**average %AA**	**EC50 FRAP**	**EC50 FRAP stdev**
**LDH-OLE_Zn-Al_1-1**	10	1.54938	1.53632	1.53	1.538566667	2.370510397	3.193446755	3.59168242	4.317993367	**39.4221194**	**2.156438875**
	20	1.43718	1.4382	1.44024	1.43854	9.440453686	9.376181474	9.247637051	10.53855721	38.74712702	
	30	1.39536	1.3668	1.33416	1.36544	12.07561437	13.87523629	15.93194707	15.08457711	41.83531248	
	40	1.224	1.26378	1.2291	1.24389	22.87334594	20.36672968	22.55198488	22.64365672	37.6839187	
		1	2	3							
**LDH-OLE_Zn-Al_2-1**	10	1.511	1.532	1.544	1.529	4.788909893	3.465658475	2.709514808	4.912935323	**52.70522117**	**5.009838843**
	20	1.409	1.439	1.427	1.425	11.21613106	9.325771897	10.08191556	11.38059701	58.06030692	
	30	1.377	1.391	1.392	1.386666667	13.23251418	12.35034657	12.28733459	13.76451078	51.92261302	
	40	1.321	1.312	1.297	1.31	16.76118463	17.32829238	18.27347196	18.53233831	48.13274356	
		1	2	3							
**LDH-OLE_Zn-Al_3-1**	10	1.51668	1.50136	1.532	1.51668	4.43100189	5.396345306	3.465658475	5.679104478	**98.67555556**	**4.956944001**
	20	1.50975	1.4945	1.525	1.50975	4.867674858	5.828607435	3.906742281	6.110074627	99.29555556	
	30	1.46124	1.44648	1.476	1.46124	7.924385633	8.854442344	6.994328922	9.126865672	93.43777778	
	40	1.46322	1.44844	1.478	1.46322	7.799621928	8.730938878	6.868304978	9.003731343	103.2933333	
		1	2	3							
**A0**		1.647	1.629	1.636	1.637333333						
**OLE**	10	1.561	1.556	1.55	1.555666667	1.638311279	1.953371141	2.331442974	4.987785016	**41.9636994**	**1.372036196**
	20	1.452	1.478	1.467	1.465666667	8.506616257	6.868304978	7.561436673	10.48452769	42.16996062	
	30	1.379	1.387	1.383	1.383	13.10649023	12.60239445	12.85444234	15.53338762	43.2209274	
	40	1.276	1.283	1.253	1.270666667	19.59672338	19.15563957	21.04599874	22.39413681	40.50021017	

Figure S4. Calculated from the Table S3 linear plots (in triplicate) which are used for the calculation of average values of $EC_{50,FRAP}$ for each tested sample.

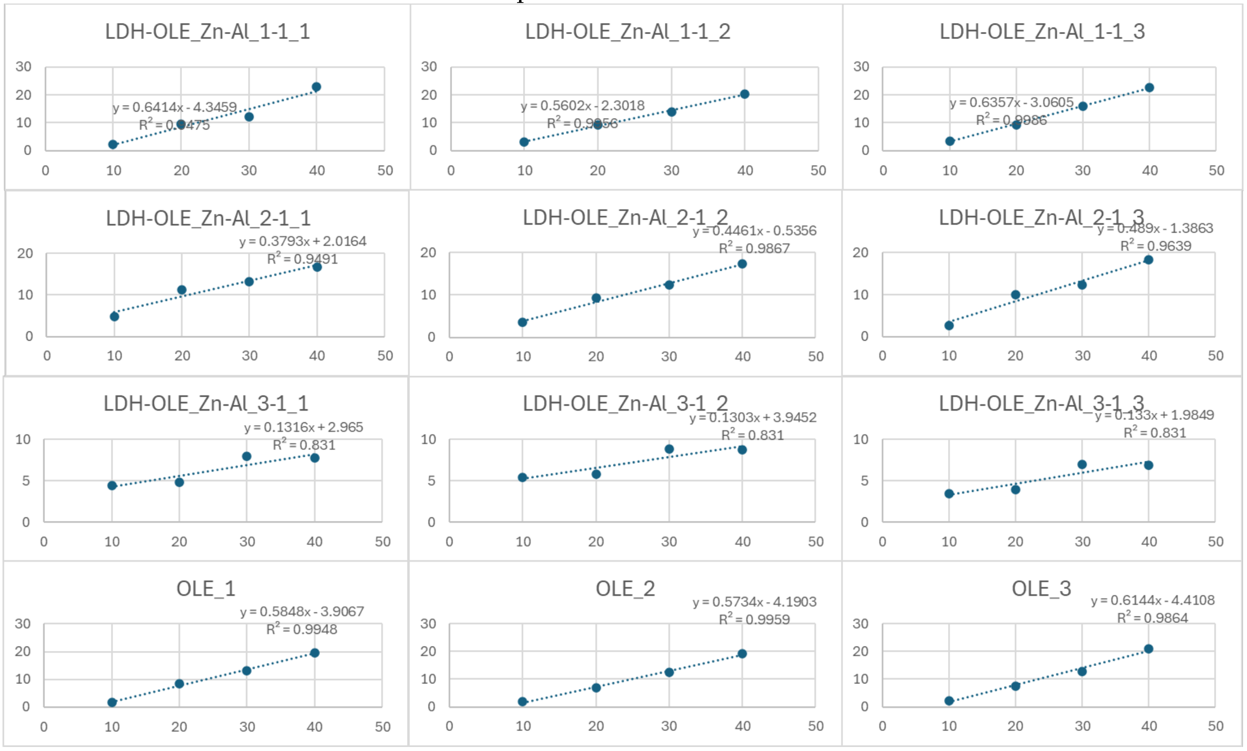

.

E-coli													
		LDH-OLE (mg/mL)											
		1	2	3	4	5	6	7	8	9	10	11	12
1/1	A	25.0	12.50	6.25	3.13	1.56	0.78	0.39	0.20	0.10	0.05	Culture	Media
	B	25.0	12.50	6.25	3.13	1.56	0.78	0.39	0.20	0.10	0.05	Culture	Media
	C	25.0	12.50	6.25	3.13	1.56	0.78	0.39	0.20	0.10	0.05	Culture	Media
2/1	D	25.0	12.50	6.25	3.13	1.56	0.78	0.39	0.20	0.10	0.05	Culture	Media
	E	25.0	12.50	6.25	3.13	1.56	0.78	0.39	0.20	0.10	0.05	Culture	Media
	F	25.0	12.50	6.25	3.13	1.56	0.78	0.39	0.20	0.10	0.05	Culture	Media
3/1	G	25.0	12.50	6.25	3.13	1.56	0.78	0.39	0.20	0.10	0.05	Culture	Media
	H	25.0	12.50	6.25	3.13	1.56	0.78	0.39	0.20	0.10	0.05	Culture	Media
	I	25.0	12.50	6.25	3.13	1.56	0.78	0.39	0.20	0.10	0.05	Culture	Media

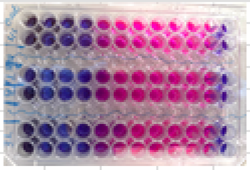

S. aureus													
		LDH-OLE (mg/mL)											
		1	2	3	4	5	6	7	8	9	10	11	12
1/1	A	25.0	12.50	6.25	3.13	1.56	0.78	0.39	0.20	0.10	0.05	Culture	Media
	B	25.0	12.50	6.25	3.13	1.56	0.78	0.39	0.20	0.10	0.05	Culture	Media
	C	25.0	12.50	6.25	3.13	1.56	0.78	0.39	0.20	0.10	0.05	Culture	Media
2/1	D	25.0	12.50	6.25	3.13	1.56	0.78	0.39	0.20	0.10	0.05	Culture	Media
	E	25.0	12.50	6.25	3.13	1.56	0.78	0.39	0.20	0.10	0.05	Culture	Media
	F	25.0	12.50	6.25	3.13	1.56	0.78	0.39	0.20	0.10	0.05	Culture	Media
3/1	G	25.0	12.50	6.25	3.13	1.56	0.78	0.39	0.20	0.10	0.05	Culture	Media
	H	25.0	12.50	6.25	3.13	1.56	0.78	0.39	0.20	0.10	0.05	Culture	Media
	I	25.0	12.50	6.25	3.13	1.56	0.78	0.39	0.20	0.10	0.05	Culture	Media

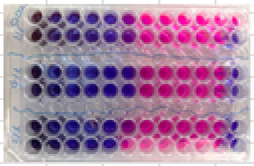

Figure S5. Results from three independent replicates for the determination of the minimum bactericidal concentration (MBC) of all OLE@LDH_Zn/Al_x/1 nanohybrids against *E. coli* and *S. aureus*. Representative images from the resazurin-based 96-well plate microdilution method used for MBC determination are also shown.

Table S4. Statistical result of $EC_{50,DPPH}$ values

Descriptives

	sample_code			Statistic	Std. Error
EC50_DPPH	OLE@LDH_Zn/Al_1/1	Mean		27,8816	1,04938
		95% Confidence Interval for Mean	Lower Bound	23,3665	
			Upper Bound	32,3967	
		5% Trimmed Mean		.	
		Median		27,0541	
		Variance		3,304	
		Std. Deviation		1,81758	
		Minimum		26,62	
		Maximum		29,97	
		Range		3,34	
		Interquartile Range		.	
		Skewness		1,624	1,225
		Kurtosis		.	.
	OLE@LDH_Zn/Al_2/1	Mean		35,6819	2,77251
		95% Confidence Interval for Mean	Lower Bound	23,7527	
			Upper Bound	47,6110	
		5% Trimmed Mean		.	
		Median		34,1677	
		Variance		23,060	
		Std. Deviation		4,80213	
		Minimum		31,82	
		Maximum		41,06	
		Range		9,24	
		Interquartile Range		.	
		Skewness		1,278	1,225
		Kurtosis		.	.
	OLE@LDH_Zn/Al_3/1	Mean		69,0668	6,06028
		95% Confidence Interval for Mean	Lower Bound	42,9915	
			Upper Bound	95,1421	
		5% Trimmed Mean		.	
		Median		70,5093	

		Variance		110,181	
		Std. Deviation		10,49672	
		Minimum		57,92	
		Maximum		78,77	
		Range		20,84	
		Interquartile Range		.	
		Skewness		-,607	1,225
		Kurtosis		.	.
	OLE	Mean		30,5582	,27736
		95% Confidence Interval for Mean	Lower Bound	29,3648	
			Upper Bound	31,7516	
		5% Trimmed Mean		.	
		Median		30,7706	
		Variance		,231	
		Std. Deviation		,48041	
		Minimum		30,01	
		Maximum		30,90	
		Range		,89	
		Interquartile Range		.	
		Skewness		-1,601	1,225
		Kurtosis		.	.

Multiple Comparisons

Dependent Variable: EC50_DPPH

Tukey HSD

(I) sample_code	(J) sample_code	Mean Difference (I-J)	Std. Error	Sig.	95% Confidence Interval	
					Lower Bound	Upper Bound
OLE@LDH_Zn/Al_1/1	OLE@LDH_Zn/Al_2/1	-7,80025	4,77452	,414	-23,0899	7,4894
	OLE@LDH_Zn/Al_3/1	-41,18518*	4,77452	<,001	-56,4749	-25,8955
	OLE	-2,67658	4,77452	,941	-17,9663	12,6131
OLE@LDH_Zn/Al_2/1	OLE@LDH_Zn/Al_1/1	7,80025	4,77452	,414	-7,4894	23,0899

	OLE@LDH_Zn/Al_3/1	-33,38493*	4,77452	<,001	-48,6746	-18,0953
	OLE	5,12367	4,77452	,714	-10,1660	20,4133
OLE@LDH_Zn/Al_3/1	OLE@LDH_Zn/Al_1/1	41,18518*	4,77452	<,001	25,8955	56,4749
	OLE@LDH_Zn/Al_2/1	33,38493*	4,77452	<,001	18,0953	48,6746
	OLE	38,50860*	4,77452	<,001	23,2189	53,7983
OLE	OLE@LDH_Zn/Al_1/1	2,67658	4,77452	,941	-12,6131	17,9663
	OLE@LDH_Zn/Al_2/1	-5,12367	4,77452	,714	-20,4133	10,1660
	OLE@LDH_Zn/Al_3/1	-38,50860*	4,77452	<,001	-53,7983	-23,2189

*. The mean difference is significant at the 0.05 level.

Table S5. Statistical result of $EC_{50,ABTS}$ values

Descriptives

	sample_code			Statistic	Std. Error
EC50_ABTS	OLE@LDH_Zn/Al_1/1	Mean		25,6961	
		95% Confidence Interval for Mean	Lower Bound	23,8097	
			Upper Bound	27,5825	
		5% Trimmed Mean		.	
		Median		25,8995	
		Variance		,577	
		Std. Deviation		,75938	
		Minimum		24,86	
		Maximum		26,33	
		Range		1,48	
		Interquartile Range		.	
		Skewness		-1,119	
		Kurtosis		.	.
	OLE@LDH_Zn/Al_2/1	Mean		35,5332	
		95% Confidence Interval for Mean	Lower Bound	27,4846	

			Upper Bound	43,5818	
		5% Trimmed Mean		.	
		Median		35,0545	
		Variance		10,498	
		Std. Deviation		3,24001	
		Minimum		32,56	
		Maximum		38,99	
		Range		6,43	
		Interquartile Range		.	
		Skewness		,650	1,225
		Kurtosis		.	.
	OLE@LDH_Zn/Al_3/1	Mean		67,8226	4,95931
		95% Confidence Interval for Mean	Lower Bound	46,4845	
			Upper Bound	89,1608	
		5% Trimmed Mean		.	
		Median		72,2357	
		Variance		73,784	
		Std. Deviation		8,58978	
		Minimum		57,92	
		Maximum		73,31	
		Range		15,39	
		Interquartile Range		.	
		Skewness		-1,702	1,225
		Kurtosis		.	.
	OLE	Mean		28,2496	,52831
		95% Confidence Interval for Mean	Lower Bound	25,9764	
			Upper Bound	30,5227	
		5% Trimmed Mean		.	
		Median		28,3707	
		Variance		,837	
		Std. Deviation		,91506	
		Minimum		27,28	

	Maximum	29,10	
	Range	1,82	
	Interquartile Range	.	
	Skewness	-,585	1,225
	Kurtosis	.	.

Multiple Comparisons

Dependent Variable: EC50_ABTS

Tukey HSD

(I) sample_code	(J) sample_code	Mean Difference (I-J)	Std. Error	Sig.	95% Confidence Interval Lower Bound	Upper Bound
OLE@LDH_Zn/Al_1/1	OLE@LDH_Zn/Al_2/1	-9,83710	3,77924	,116	-21,9396	2,2654
	OLE@LDH_Zn/Al_3/1	-42,12653*	3,77924	<.001	-54,2290	-30,0241
	OLE	-2,55346	3,77924	,903	-14,6559	9,5490
OLE@LDH_Zn/Al_2/1	OLE@LDH_Zn/Al_1/1	9,83710	3,77924	,116	-2,2654	21,9396
	OLE@LDH_Zn/Al_3/1	-32,28943*	3,77924	<.001	-44,3919	-20,1870
	OLE	7,28364	3,77924	,290	-4,8188	19,3861
OLE@LDH_Zn/Al_3/1	OLE@LDH_Zn/Al_1/1	42,12653*	3,77924	<.001	30,0241	54,2290
	OLE@LDH_Zn/Al_2/1	32,28943*	3,77924	<.001	20,1870	44,3919
	OLE	39,57307*	3,77924	<.001	27,4706	51,6755
OLE	OLE@LDH_Zn/Al_1/1	2,55346	3,77924	,903	-9,5490	14,6559
	OLE@LDH_Zn/Al_2/1	-7,28364	3,77924	,290	-19,3861	4,8188
	OLE@LDH_Zn/Al_3/1	-39,57307*	3,77924	<.001	-51,6755	-27,4706

*. The mean difference is significant at the 0.05 level.

Table S6. Statistical result of $EC_{50,FRAP}$ values

Descriptives

	sample_code			Statistic	Std. Error
**EC50_FRAP**	**OLE@LDH_Zn/Al_1/1**	**Mean**		**39,4221**	**1,24502**
		**95% Confidence Interval for Mean**	**Lower Bound**	**34,0652**	

			Upper Bound	44,7790	
		5% Trimmed Mean		.	
		Median		38,7471	
		Variance		4,650	
		Std. Deviation		2,15644	
		Minimum		37,68	
		Maximum		41,84	
		Range		4,15	
		Interquartile Range		.	
		Skewness		1,271	1,225
		Kurtosis		.	.
	OLE@LDH_Zn/Al_2/1	Mean		52,7052	2,89243
		95% Confidence Interval for Mean	Lower Bound	40,2601	
			Upper Bound	65,1504	
		5% Trimmed Mean		.	
		Median		51,9226	
		Variance		25,098	
		Std. Deviation		5,00984	
		Minimum		48,13	
		Maximum		58,06	
		Range		9,93	
		Interquartile Range		.	
		Skewness		,686	1,225
		Kurtosis		.	.
	OLE@LDH_Zn/Al_3/1	Mean		98,6756	2,86189
		95% Confidence Interval for Mean	Lower Bound	86,3618	
			Upper Bound	110,9893	
		5% Trimmed Mean		.	
		Median		99,2956	
		Variance		24,571	
		Std. Deviation		4,95694	
		Minimum		93,44	

		Maximum		103,29	
		Range		9,86	
		Interquartile Range		.	
		Skewness		-,554	1,225
		Kurtosis		.	.
	OLE	Mean		41,9637	,79215
		95% Confidence Interval for Mean	Lower Bound	38,5554	
			Upper Bound	45,3720	
		5% Trimmed Mean		.	
		Median		42,1700	
		Variance		1,882	
		Std. Deviation		1,37204	
		Minimum		40,50	
		Maximum		43,22	
		Range		2,72	
		Interquartile Range		.	
		Skewness		-,661	1,225
		Kurtosis		.	.

Multiple Comparisons

Dependent Variable: EC50_FRAP

Tukey HSD

					95% Confidence Interval	
(I) sample_code	(J) sample_code	Mean Difference (I-J)	Std. Error	Sig.	Lower Bound	Upper Bound
OLE@LDH_Zn/Al_1/1	OLE@LDH_Zn/Al_2/1	-13,28310*	3,06057	,011	-23,0841	-3,4821
	OLE@LDH_Zn/Al_3/1	-59,25344*	3,06057	<.001	-69,0545	-49,4524
	OLE	-2,54158	3,06057	,839	-12,3426	7,2594
OLE@LDH_Zn/Al_2/1	OLE@LDH_Zn/Al_1/1	13,28310*	3,06057	,011	3,4821	23,0841
	OLE@LDH_Zn/Al_3/1	-45,97033*	3,06057	<.001	-55,7714	-36,1693
	OLE	10,74152*	3,06057	,033	,9405	20,5425
OLE@LDH_Zn/Al_3/1	OLE@LDH_Zn/Al_1/1	59,25344*	3,06057	<.001	49,4524	69,0545
	OLE@LDH_Zn/Al_2/1	45,97033*	3,06057	<.001	36,1693	55,7714
	OLE	56,71186*	3,06057	<.001	46,9108	66,5129

**OLE**	**OLE@LDH_Zn/Al_1/1**	**2,54158**	**3,06057**	**,839**	**-7,2594**	**12,3426**
	**OLE@LDH_Zn/Al_2/1**	**-10,74152***	**3,06057**	**,033**	**-20,5425**	**-,9405**
	**OLE@LDH_Zn/Al_3/1**	**-56,71186***	**3,06057**	**<.001**	**-66,5129**	**-46,9108**

***. The mean difference is significant at the 0.05 level.**

Table S7. Statistical result of TPC values

Descriptives

	sample_code			Statistic	Std. Error
TPC_pH7	OLE@LDH_Zn/Al_1/1	Mean		21,6296	3,12766
		95% Confidence Interval for Mean	Lower Bound	8,1724	
			Upper Bound	35,0868	
		5% Trimmed Mean		.	
		Median		19,2222	
		Variance		29,347	
		Std. Deviation		5,41726	
		Minimum		17,83	
		Maximum		27,83	
		Range		10,00	
		Interquartile Range		.	
		Skewness		1,605	1,225
		Kurtosis		.	.
	OLE@LDH_Zn/Al_2/1	Mean		23,8519	2,82521
		95% Confidence Interval for Mean	Lower Bound	11,6959	
			Upper Bound	36,0078	
		5% Trimmed Mean		.	
		Median		21,1667	
		Variance		23,945	
		Std. Deviation		4,89341	
		Minimum		20,89	
		Maximum		29,50	
		Range		8,61	
		Interquartile Range		.	
		Skewness		1,726	1,225
		Kurtosis		.	.
	OLE@LDH_Zn/Al_3/1	Mean		28,5741	4,78534
		95% Confidence Interval for Mean	Lower Bound	7,9844	
			Upper Bound	49,1637	

		5% Trimmed Mean		.	
		Median		24,5000	
		Variance		68,699	
		Std. Deviation		8,28846	
		Minimum		23,11	
		Maximum		38,11	
		Range		15,00	
		Interquartile Range		.	
		Skewness		1,677	1,225
		Kurtosis		.	.
	OLE	Mean		646,4444	1,60375
		95% Confidence Interval for Mean	Lower Bound	639,5441	
			Upper Bound	653,3448	
		5% Trimmed Mean		.	
		Median		646,4444	
		Variance		7,716	
		Std. Deviation		2,77778	
		Minimum		643,67	
		Maximum		649,22	
		Range		5,56	
		Interquartile Range		.	
		Skewness		,000	1,225
		Kurtosis		.	.
TPC_pH3.6	OLE@LDH_Zn/Al_1/1	Mean		25,0556	,57824
		95% Confidence Interval for Mean	Lower Bound	22,5676	
			Upper Bound	27,5435	
		5% Trimmed Mean		.	
		Median		24,7778	
		Variance		1,003	
		Std. Deviation		1,00154	
		Minimum		24,22	
		Maximum		26,17	
		Range		1,94	
		Interquartile Range		.	
		Skewness		1,152	1,225
		Kurtosis		.	.
	OLE@LDH_Zn/Al_2/1	Mean		40,1481	1,06783
		95% Confidence Interval for Mean	Lower Bound	35,5536	

			Upper Bound	44,7426	
		5% Trimmed Mean		.	
		Median		40,6111	
		Variance		3,421	
		Std. Deviation		1,84954	
		Minimum		38,11	
		Maximum		41,72	
		Range		3,61	
		Interquartile Range		.	
		Skewness		-1,056	1,225
		Kurtosis		.	.
	OLE@LDH_Zn/Al_3/1	Mean		22,7407	,37037
		95% Confidence Interval for Mean	Lower Bound	21,1472	
			Upper Bound	24,3343	
		5% Trimmed Mean		.	
		Median		23,1111	
		Variance		,412	
		Std. Deviation		,64150	
		Minimum		22,00	
		Maximum		23,11	
		Range		1,11	
		Interquartile Range		.	
		Skewness		-1,732	1,225
		Kurtosis		.	.
	OLE	Mean		643,6667	1,60375
		95% Confidence Interval for Mean	Lower Bound	636,7663	
			Upper Bound	650,5670	
		5% Trimmed Mean		.	
		Median		643,6667	
		Variance		7,716	
		Std. Deviation		2,77778	
		Minimum		640,89	
		Maximum		646,44	
		Range		5,56	
		Interquartile Range		.	
		Skewness		,000	1,225
		Kurtosis		.	.
TPC_pH1	OLE@LDH_Zn/Al_1/1	Mean		606,6296	4,03602

		95% Confidence Interval for Mean	Lower Bound	589,2640	
			Upper Bound	623,9952	
		5% Trimmed Mean		.	
		Median		607,5556	
		Variance		48,868	
		Std. Deviation		6,99059	
		Minimum		599,22	
		Maximum		613,11	
		Range		13,89	
		Interquartile Range		.	
		Skewness		-,586	1,225
		Kurtosis		.	.
	OLE@LDH_Zn/Al_2/1	Mean		232,0926	4,34594
		95% Confidence Interval for Mean	Lower Bound	213,3935	
			Upper Bound	250,7917	
		5% Trimmed Mean		.	
		Median		232,8333	
		Variance		56,662	
		Std. Deviation		7,52738	
		Minimum		224,22	
		Maximum		239,22	
		Range		15,00	
		Interquartile Range		.	
		Skewness		-,439	1,225
		Kurtosis		.	.
	OLE@LDH_Zn/Al_3/1	Mean		186,9074	8,81966
		95% Confidence Interval for Mean	Lower Bound	148,9595	
			Upper Bound	224,8553	
		5% Trimmed Mean		.	
		Median		181,1667	
		Variance		233,359	
		Std. Deviation		15,27609	
		Minimum		175,33	
		Maximum		204,22	
		Range		28,89	
		Interquartile Range		.	
		Skewness		1,452	1,225
		Kurtosis		.	.

OLE	Mean		634,4074	2,44977
	95% Confidence Interval for Mean	Lower Bound	623,8669	
		Upper Bound	644,9479	
	5% Trimmed Mean		.	
	Median		635,3333	
	Variance		18,004	
	Std. Deviation		4,24313	
	Minimum		629,78	
	Maximum		638,11	
	Range		8,33	
	Interquartile Range		.	
	Skewness		-,935	1,225
	Kurtosis		.	.

Multiple Comparisons

Tukey HSD

Dependent Variable	(I) sample_code	(J) sample_code	Mean Difference (I-J)	Std. Error	Sig.	95% Confidence Interval Lower Bound	95% Confidence Interval Upper Bound
TPC_pH1	OLE@LDH_Zn/Al_1/1	OLE@LDH_Zn/Al_2/1	374,53704*	7,71247	<.001	349,8390	399,2351
		OLE@LDH_Zn/Al_3/1	419,72222*	7,71247	<.001	395,0242	444,4203
		OLE	-27,77778*	7,71247	,029	-52,4758	-3,0797
	OLE@LDH_Zn/Al_2/1	OLE@LDH_Zn/Al_1/1	-374,53704*	7,71247	<.001	-399,2351	-349,8390
		OLE@LDH_Zn/Al_3/1	45,18519*	7,71247	,002	20,4871	69,8832
		OLE	-402,31481*	7,71247	<.001	-427,0129	-377,6168
	OLE@LDH_Zn/Al_3/1	OLE@LDH_Zn/Al_1/1	-419,72222*	7,71247	<.001	-444,4203	-395,0242
		OLE@LDH_Zn/Al_2/1	-45,18519*	7,71247	,002	-69,8832	-20,4871
		OLE	-447,50000*	7,71247	<.001	-472,1980	-422,8020
	OLE	OLE@LDH_Zn/Al_1/1	27,77778*	7,71247	,029	3,0797	52,4758
		OLE@LDH_Zn/Al_2/1	402,31481*	7,71247	<.001	377,6168	427,0129
		OLE@LDH_Zn/Al_3/1	447,50000*	7,71247	<.001	422,8020	472,1980
TPC_pH3.6	OLE@LDH_Zn/Al_1/1	OLE@LDH_Zn/Al_2/1	-15,09259*	1,44634	<.001	-19,7243	-10,4609
		OLE@LDH_Zn/Al_3/1	2,31481	1,44634	,430	-2,3169	6,9465
		OLE	-618,61111*	1,44634	<.001	-623,2428	-613,9794
	OLE@LDH_Zn/Al_2/1	OLE@LDH_Zn/Al_1/1	15,09259*	1,44634	<.001	10,4609	19,7243
		OLE@LDH_Zn/Al_3/1	17,40741*	1,44634	<.001	12,7757	22,0391

		OLE	-603,51852*	1,44634	<.001	-608,1502	-598,8868
	OLE@LDH_Zn/Al_3/1	OLE@LDH_Zn/Al_1/1	-2,31481	1,44634	,430	-6,9465	2,3169
		OLE@LDH_Zn/Al_2/1	-17,40741*	1,44634	<.001	-22,0391	-12,7757
		OLE	-620,92593*	1,44634	<.001	-625,5576	-616,2942
	OLE	OLE@LDH_Zn/Al_1/1	618,61111*	1,44634	<.001	613,9794	623,2428
		OLE@LDH_Zn/Al_2/1	603,51852*	1,44634	<.001	598,8868	608,1502
		OLE@LDH_Zn/Al_3/1	620,92593*	1,44634	<.001	616,2942	625,5576
TPC_pH7	OLE@LDH_Zn/Al_1/1	OLE@LDH_Zn/Al_2/1	-2,22222	4,64949	,962	-17,1115	12,6671
		OLE@LDH_Zn/Al_3/1	-6,94444	4,64949	,483	-21,8338	7,9449
		OLE	-624,81481*	4,64949	<.001	-639,7041	-609,9255
	OLE@LDH_Zn/Al_2/1	OLE@LDH_Zn/Al_1/1	2,22222	4,64949	,962	-12,6671	17,1115
		OLE@LDH_Zn/Al_3/1	-4,72222	4,64949	,745	-19,6115	10,1671
		OLE	-622,59259*	4,64949	<.001	-637,4819	-607,7033
	OLE@LDH_Zn/Al_3/1	OLE@LDH_Zn/Al_1/1	6,94444	4,64949	,483	-7,9449	21,8338
		OLE@LDH_Zn/Al_2/1	4,72222	4,64949	,745	-10,1671	19,6115
		OLE	-617,87037*	4,64949	<.001	-632,7597	-602,9811
	OLE	OLE@LDH_Zn/Al_1/1	624,81481*	4,64949	<.001	609,9255	639,7041
		OLE@LDH_Zn/Al_2/1	622,59259*	4,64949	<.001	607,7033	637,4819
		OLE@LDH_Zn/Al_3/1	617,87037*	4,64949	<.001	602,9811	632,7597

*. The mean difference is significant at the 0.05 level.
